# Prp43 Bound at Different Sites on the Pre-rRNA Performs Distinct Functions in Ribosome Synthesis

**DOI:** 10.1016/j.molcel.2009.09.039

**Published:** 2009-11-25

**Authors:** Markus T. Bohnsack, Roman Martin, Sander Granneman, Maike Ruprecht, Enrico Schleiff, David Tollervey

**Affiliations:** 1 Wellcome Trust Centre for Cell Biology, University of Edinburgh, Edinburgh EH9 3JR, UK; 2 Centre for Systems Biology, University of Edinburgh, Edinburgh EH9 3JR, UK; 3 Cluster of Excellence Macromolecular Complexes, Institute for Molecular Biosciences, Goethe University, Max-von-Laue-Strasse 9, 60438 Frankfurt, Germany; 4 Centre of Membrane Proteomics, Molecular Cell Biology, Goethe University, Max-von-Laue-Strasse 9, 60438 Frankfurt, Germany

**Keywords:** PROTEINS, RNA

## Abstract

Yeast ribosome synthesis requires 19 different RNA helicases, but none of their pre-rRNA-binding sites were previously known, making their precise functions difficult to determine. Here we identify multiple binding sites for the helicase Prp43 in the 18S and 25S rRNA regions of pre-rRNAs, using UV crosslinking. Binding in 18S was predominantly within helix 44, close to the site of 18S 3′ cleavage, in which Prp43 is functionally implicated. Four major binding sites were identified in 25S, including helix 34. In strains depleted of Prp43 or expressing only catalytic point mutants, six snoRNAs that guide modifications close to helix 34 accumulated on preribosomes, implicating Prp43 in their release, whereas other snoRNAs showed reduced preribosome association. Prp43 was crosslinked to snoRNAs that target sequences close to its binding sites, indicating direct interactions. We propose that Prp43 acts on preribosomal regions surrounding each binding site, with distinct functions at different locations.

## Introduction

Ribosomes in *Saccharomyces cerevisiae* consist of 79 ribosomal proteins and four ribosomal RNAs (rRNAs). The 25S, 18S, and 5.8S rRNAs are derived from a primary transcript, the 35S preribosomal RNA (pre-rRNA), via multiple cleavage, trimming, and modification events, while the 5S rRNA is transcribed independently (reviewed in Venema and Tollervey, 1999; Fromont-Racine et al., 2003; Tschochner and Hurt, 2003; Granneman and Baserga, 2004; Henras et al., 2008). Early cleavages at sites A_0_–A_2_ take place in a large complex termed the SSU processome or 90S preribosome, and processing at these sites separates the biogenesis pathways of the two ribosomal subunits. The pre-rRNA intermediates of the pre-60S complexes are processed in the nucleus before nuclear export to generate the mature rRNAs, while the final cleavage step in 18S synthesis, cleavage at site D in the 20S pre-rRNA, occurs in the cytoplasm.

Most nucleotide modifications in the rRNA are 2′-O-methylation and pseudouridylation at sites selected by base pairing with box C/D and box H/ACA small nucleolar RNAs (snoRNAs), respectively (Kiss-Laszlo et al., 1996; Ganot et al., 1997; Bachellerie et al., 2002). Seventy-two modification guide snoRNAs are individually nonessential, although combinations of snoRNA deletions can lead to a lethal phenotype (Kiss, 2002). Other snoRNAs are essential for viability and for the early pre-rRNA cleavages at the sites A_0_–A_2_: the box C/D snoRNAs U3 and U14, and the box H/ACA snoRNA snR30 (Li et al., 1990; Hughes and Ares, 1991; Beltrame and Tollervey, 1995; Liang and Fournier, 1995; Morrissey and Tollervey, 1997). In general, the base pairing of the pre-rRNA with box H/ACA snoRNAs is predicted to be less stable than for box C/D snoRNAs, which have long complementary sequences of up to 19 nucleotides. However, both classes of snoRNA can be detected in stable association with 60–90S preribosomes on sucrose density gradients.

Besides the components of the small nucleolar ribonucleoprotein particles (snoRNPs), more than 180 nonribosomal cofactors are involved in the ribosome biogenesis pathway. These include exonucleases and endonucleases involved in pre-rRNA processing, as well as GTPases, kinases, and RNA helicases (see, for example, Fromont-Racine et al., 2003; Henras et al., 2008). Nineteen putative RNA helicases are known to function in ribosome biogenesis in *Saccharomyces cerevisiae*. All of these are designated as DEAD/H or DExD box proteins with the exception of the DEVH box helicase Dob1/Mtr4, a cofactor of the exosome complex required for 3′ maturation of the 5.8S rRNA (de la Cruz et al., 1998; Linder, 2006). Dbp4, Has1, and Rok1 were shown to be required for release of small numbers of specific snoRNAs from preribosomes (Kos and Tollervey, 2005; Liang and Fournier, 2006; Bohnsack et al., 2008), but for no helicase was the binding site(s) on the preribosomes known. The molecular functions of all other RNA helicases acting in ribosome biogenesis have remained elusive so far. It seemed possible that other helicases would also act to displace snoRNPs from preribosomes, but attempts to demonstrate this by screening all 75 snoRNAs in a panel of mutants were largely unsuccessful (Bohnsack et al., 2008).

Based on defects observed in RNA processing following genetic depletion, most putative helicases are predicted to act during either 40S or 60S ribosome subunit synthesis: Dbp4, Dbp8, Dhr1, Dhr2, Fal1, Rok1, and Rrp3 are required for 20S pre-rRNA synthesis and 40S biogenesis, whereas Dbp2, Dbp3, Dbp6, Dbp7, Dbp9, Dbp10, Drs1, Mak5, and Spb4 are required for 27S pre-rRNA processing and 60S biogenesis (Rocak and Linder, 2004; Bleichert and Baserga 2007; Cordin et al., 2006 and references therein). However, Has1 and the DEAH box helicase Prp43 are implicated in both the 40S and the 60S biogenesis pathways (Emery et al., 2004; Lebaron et al., 2005).

Prp43 was initially identified as a pre-mRNA splicing factor acting in the release of the intron lariat from the spliceosome (Arenas and Abelson, 1997; Martin et al., 2002; Tanaka et al., 2007) but was subsequently shown to function in ribosome synthesis (Lebaron et al., 2005; Combs et al., 2006; Leeds et al., 2006). Depletion of Prp43 results in accumulation of 35S pre-rRNA, combined with a reduction in the levels of downstream pre-rRNA intermediates and mature rRNAs. Tandem affinity purification (TAP) with tagged Prp43 precipitated pre-rRNAs including the 20S and 27S precursors, indicating its involvement in both 40S and 60S subunit synthesis. Genetic analyses implicated Prp43 in cleavage of the 20S pre-rRNA that generates the 3′ end of the 18S rRNA (Pertschy et al., 2009). However, the binding sites and molecular function of Prp43 remained unclear.

Here, we used an unbiased crosslinking and cDNA analysis (CRAC) approach to identify binding sites of Prp43 on the pre-rRNA—and find complex results. In line with previous coprecipitation analyses, we identify major binding sites in both the 18S and the 25S regions of the pre-rRNA. Our results are consistent with the proposed role for Prp43 in site D cleavage but also imply that Prp43 is required to displace a subset of box C/D snoRNAs from pre-66S complexes. Thus Prp43 fulfills distinct functions in the biogenesis pathways of the small and the large ribosomal subunit.

## Results

### Prp43 Binds to Distinct Sites on Ribosomal RNA

Prp43 and Rok1 were previously reported to function in ribosome biogenesis (Venema et al., 1997; Lebaron et al., 2005; Combs et al., 2006; Leeds et al., 2006). Rok1 was implicated in release of snR30 from preribosomes (Bohnsack et al., 2008), but its interaction site(s) with any of the RNAs remained unknown. For Prp43, neither its interaction sites with pre-rRNA nor its molecular function in the pathway had been identified. In order to locate binding sites for Prp43 and Rok1 on RNA, we made use of the CRAC UV crosslinking method (Granneman et al., 2009). *PRP43* or *ROK1* were genomically tagged with a C-terminal His_6_-TEV-ProteinA (HTP) tag, allowing a first purification step on IgG Sepharose, elution by cleavage with tobacco etch virus protease (TEV), and final purification of protein-RNA complexes on nickel beads under denaturing conditions. Rok1 was not detectably UV crosslinked to RNA, perhaps reflecting very transient interactions with the pre-rRNA or recruitment that predominately involves protein factors. In contrast, Prp43 crosslinking to RNA was readily detectable by radiolabeling of crosslinked RNA (see Figure S1A available online). UV crosslinking was performed either in vivo in living cells on ice or in vitro after TEV elution of Prp43 containing complexes from IgG beads. RNA molecules crosslinked to Prp43-HTP were partially digested with RNase A + T1, ligated to linkers, and amplified by RT-PCR. Binding sites were identified either by cloning and Sanger sequencing or direct Solexa sequencing of PCR products. The resulting sequences were assigned to genomic locations by NOVOALIGN (http://www.novocraft.com/) (Granneman et al., 2009) and grouped into functional categories (see Table 1, Figure S1).

Interestingly, in vivo CRAC with cells expressing Prp43-HTP (Table 1, Figure S1B) resulted in 50-fold enrichment of box C/D snoRNAs as compared to the control strain (Table 1, Figure S1D), while box H/ACA snoRNAs were enriched less than 4-fold. No significant enrichment of intron-containing genes was observed, whereas snRNAs were enriched 17-fold. In both the Prp43-HTP and parental strain, almost 75% of all sequences were of ribosomal origin. Ninety-eight percent of sequences derived from in vitro crosslinked Prp43-containing complexes were rRNA sequences (Table 1, Figure S1C), suggesting that more transient interactions were lost during cell lysis and complex enrichment.

**Figure 1:**
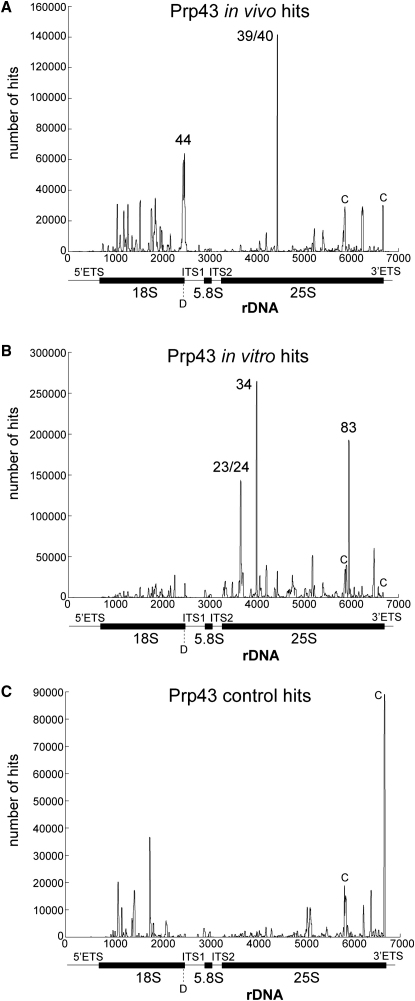
Identification of Putative Binding Sites of Prp43 on Ribosomal RNA Control cells (C) or Prp43-HTP-expressing cells (A) were UV crosslinked in vivo or crosslinking was performed in vitro after cell lysis and enrichment of Prp43-HTP-containing complexes on IgG Sepharose (B). Crosslinked RNAs were trimmed and ligated to linkers followed by RT-PCR and Solexa sequencing. The obtained sequences were aligned with the rDNA encoding 35S pre-rRNA (nucleotides 1–6880), and the number of hits for each individual nucleotide is plotted. The position of the mature 18S, 5.8S, and 25S rRNAs is indicated by bars below, and processing site D is shown. Numbers at peaks indicate the corresponding helices of 18S or 25S, while 25S peaks labeled with “C” represent background peaks also found in the control. In vivo crosslinking of Prp43-HTP-expressing cells resulted in two major peaks, whereas in vitro crosslinking of purified complexes yielded three sequence clusters. Thus, CRAC experiments have identified several potential binding sites for Prp43 on pre-rRNA.

**Figure 2:**
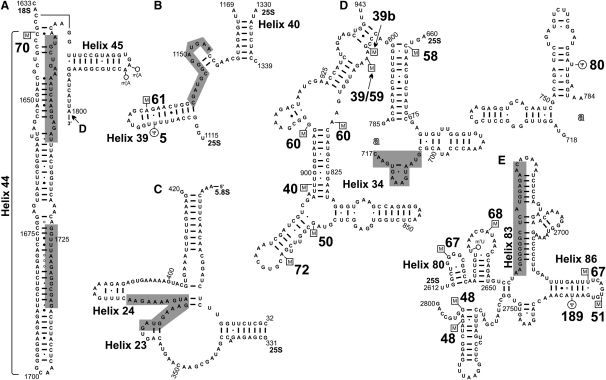
Prp43 Has Distinct Binding Sites on Preribosomal RNA Shown is predicted secondary structure of regions of yeast 18S (A) and 25S rRNA (B–E), modified from Piekna-Przybylska et al. (2007). The binding sites of Prp43 are marked in gray. A boxed “M” represents a site of 2′-O-methylation in the rRNA, and large numbers indicate the corresponding snoRNA. Small numbers represent the position of the indicated residue in 18S or 25S. In (C), part of the 25S structure is excised at “a” and shown below the main model, as it projects into the background. The major binding site for Prp43 in 18S is located in helix 44 (A), close to the dimethylation sites of Dim1 ($m_{2}^{6}$A$m_{2}^{6}$A) and cleavage site D (the 3′ end of the 18S rRNA). In 25S, Prp43 binds between helices 39 and 40 (B), between helices 23 and 24 (C), over helix 34 (D), and along helix 83 (E).

Ribosomal sequences obtained by CRAC were aligned against the rDNA, and the number of hits was determined for each individual nucleotide. Peaks represent clusters of hits covering the same sequence and are therefore potential binding sites for Prp43 (Figure 1). In total, five major peaks were identified by in vivo and in vitro crosslinking (see the supplemental sequence alignments in the Supplemental Data). In addition, multiple smaller clusters were found, some of which were also present in the control. A very similar distribution of hits was obtained by cloning and Sanger sequencing of the RT-PCR products (data not shown). In vivo crosslinking of Prp43-HTP enriched two major regions relative to the control (Figure 1A). A bipartite peak was observed over rDNA nucleotides 2440 and 2470, corresponding to sites within helix 44 near the 3′ end of 18S (Figure 2A). These sites are located close to pre-rRNA cleavage site D, where processing of the 20S pre-rRNA to the mature 18S rRNA occurs. This observation is consistent with the model that Prp43 acts to promote pre-rRNA cleavage at site D by the endonuclease Nob1 (Pertschy et al., 2009). One site of RNA 2′-O-methylation is located within this region, which is directed by snR70 (Figure 2A). The other major in vivo peak was located over rDNA nucleotides 4427-4440, which was centered at 25S+1145, between helices 39 and 40. This 25S peak is located close to the 2′-O-methylation site (Am1133) directed by the box C/D snoRNP snR61 and to the pseudouridylation site (Ψ1124) directed by box H/ACA snoRNP snR5 (Figure 2B).

In vitro crosslinking identified three major binding sites for Prp43 in the 25S rRNA region (Figure 1B). Crosslinking seen over rDNA nucleotides 3648–3664 was centered at 25S+370 between helices 23 and 24. This site lies within the region of interaction between the 25S and 5.8S rRNAs, which are stably base paired in 60S ribosomes (Figure 2C). No sites of snoRNA-directed rRNA modification are located in this region. The highest peak of Prp43 sequence reads was over rDNA nucleotides 3988–4002, centered at 25S+710 in helix 34 (Figure 2D). This site lies close to a cluster of 2′-O-methylation sites directed by box C/D snoRNPs snR39 (Am807), snR39b (Gm805), snR40 (Um898), snR50 (Gm867), snR58 (Cm663), snR59 (Am807), snR60 (Am817, Gm908), and snR72 (Am876), and the box H/ACA snoRNA snR80 (Psi776) (Figure 2D). Crosslinks over nucleotides 5941–5960 (centered at 25S+2680 in helix 83) (Figure 2E) also lie close to sites of modification by snR48 (Gm2791, Gm2793), snR51 (Um2729), snR67 (Gm2619, Um2724), snR68 (Am2640), and snR189 (Psi2735).

We conclude that Prp43 has several distinct interaction sites on pre-rRNA. The binding site at helix 44 in the 18S rRNA is close to a site of pre-rRNA cleavage, in which Prp43 is implicated. In contrast, the 25S rRNA crosslinks are not predicted to lie close to processing sites, but the helix 39/40 crosslink lies close to rRNA modification sites, while helixes 34 and 83 are located close to prominent clusters of box C/D snoRNP-directed modification sites. Notable differences were observed in the major sites of crosslinking in vivo and in vitro. These presumably reflect differences in residence time at the various sites during in vivo ribosome assembly, compared to differences in stability of binding to these sites during preribosome purification in vitro.

### Prp43 Crosslinks to a Subset of Box C/D snoRNAs and the U6 snRNA

**Figure 3:**
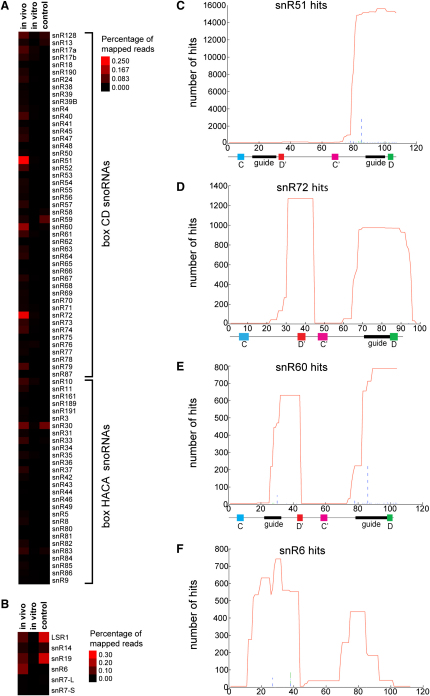
Prp43 Crosslinking to snoRNAs and snRNAs Protein-RNA complexes from Prp43-HTP-expressing cells and control cells were crosslinked and analyzed as described for Figure 1. The percentage of sequences found for individual snoRNAs (A) or snRNAs (B) and the crosslinking sites of Prp43 on the most highly enriched snoRNAs snR51 (C), snR72 (D), snR60 (E), and the splicing snRNA U6 (F) are presented as total number of hits. The total number of hits (red line) and the positions of deletions (dashed green line) and nucleotide substitutions (dashed blue line) in the obtained sequences are shown. The regions of the guide sequences and functional elements of the snoRNAs are indicated below.

Small nuclear RNA sequences obtained after in vivo crosslinking of Prp43-HTP expressing cells or controls are shown as heat maps, indicating the percentage of mapped reads for each snoRNA (Figure 3A) or snRNA (Figure 3B). In vitro crosslinking of Prp43-containing complexes did not significantly enrich for small noncoding RNAs (Table S2).

For several box C/D snoRNAs, reads were much more frequent following Prp43 in vivo CRAC than in the control experiment (Figure 3A and Table S2). snR51, snR60, and snR72 were most frequently recovered, with lower but still significant recovery of other snoRNAs including snR40 and snR61. snR51 guides methylation close to the Prp43-binding site in 25S helix 83 (Figure 2E), snR72, snR60, and snR40 guide methylation at sites close to the helix 34-binding site (Figure 2D), and snR61 binds close to the in vivo Prp43 crosslink at helices 39/40 (Figure 2B).

When the reads were mapped onto the snoRNA sequences, they clustered around the guide sequence close to box D in case of snR51 (Figure 3C), while for snR72 (Figure 3D) and snR60 (Figure 3E) an additional cluster was found at box D′, partially covering the second guide sequence of snR60 (see the supplemental sequence alignments in the Supplemental Data). Granneman et al. (2009) reported that nucleotide substitutions or deletions could reveal precise crosslinking sites. We mapped specific mutations in or close to guide sequences, strongly supporting direct contacts between Prp43 and the guide sequences of these snoRNAs.

We conclude that Prp43 directly binds both snoRNAs and regions of the pre-rRNA in close proximity to their sites of modification.

Three spliceosomal snRNAs (U2, U5, and U6) were previously reported to be enriched in Prp43 coprecipitation experiments (Lebaron et al., 2005; Combs et al., 2006; Leeds et al., 2006). Prp43 was significantly crosslinked to snR6 (U6), but not to snR7S/L (U5) or LSR1 (U2) (Figure 3B). When the reads were mapped onto the U6 sequence, two peaks of Prp43 binding were observed, over positions 18–43 and 76–83 (Figure 3F). The 3′ peak partially overlaps the U4-interaction domain (nucleotides 55–80), but no corresponding hits were enriched for U4, consistent with the proposal that Prp43 acts in spliceosome disassembly when U4 and U6 are expected to have dissociated (Arenas and Abelson, 1997; Martin et al., 2002; Tanaka et al., 2007).

### Depletion of Prp43 Traps snoRNAs in Preribosomes

Three RNA helicases were previously implicated in release of snoRNAs from preribosomes (Kos and Tollervey, 2005; Liang and Fournier, 2006; Bohnsack et al., 2008). The observation that Prp43 crosslinked to pre-rRNA regions that are heavily modified by snoRNPs prompted us to analyze whether Prp43 is required for incorporation or release of snoRNAs.

**Figure 4:**
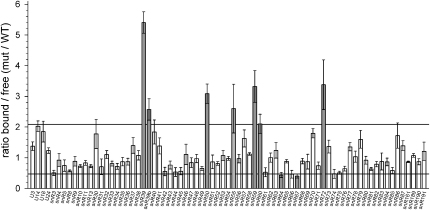
Prp43 Is Required for Release of snoRNAs from Preribosomes WT and P_tet_-*prp43* strains were pregrown in YPD medium and doxycycline was added for 6 hr to allow Prp43 depletion. Soluble cellular extracts were prepared and separated on 10%–45% sucrose gradients. RNA was extracted from gradient fractions, fractions containing either free or preribosome-bound snoRNAs were pooled, and the levels of all 75 yeast snoRNAs were determined by quantitative RT-PCR. Data from depletions were normalized to WT samples processed in parallel and average ratios of preribosome-bound to unbound snoRNAs are shown. Error bars represent standard error. snoRNAs showing an accumulation outside the 95% confidence interval (lines) are marked in gray. Depletion of Prp43 results in a shift of snR39, snR39b, snR50, snR55, snR59, snR60, and snR72 into fractions containing preribosomes, while the preribosomal levels of snR64 and snR67 are reduced.

We analyzed the effect of Prp43 depletion on the distribution of snoRNAs in sucrose density gradients, comparing free snoRNAs at the top of the gradient to fractions containing 40–90S preribosomes. Quantitative PCR (qPCR) was used to simultaneously analyze all 75 yeast snoRNAs (Bohnsack et al., 2008) in a strain that carries genomic *PRP43* under the control of the tetracycline/doxycycline repressible promoter (P_tet_-*prp43* strain). Analyses were performed 6 hr after doxycycline addition, prior to the appearance of any growth defect. The results were expressed as the ratio of preribosome bound to free snoRNA, normalized to the ratio of wild-type (WT) samples processed in parallel, and set as 1 (Figure 4).

Analysis of the effects of Prp43 depletion revealed changes in the distribution of many box C/D snoRNAs. Among snoRNAs that showed decreased abundance in preribosomal fractions, only snR64 and snR67 were reduced below the value of 0.48 (95% confidence; marked in gray). Several other snoRNAs, including snR61, were just above the 95% confidence threshold. snR67 has target sequences flanking the Prp43-binding site at helix 83 (Figure 2E), whereas snR61 targets sequences close to the Prp43-binding site at helix 39/40 (Figure 2B).

Conversely, the snoRNAs snR39, snR39b, snR50, snR55, snR59, snR60, and snR72 were found to shift from the free pool to preribosomal fractions to above the value of 2.1 (95% confidence) upon depletion of Prp43 (Figure 4, in gray), while the snoRNAs snR40 and snR70 fell just below this threshold. The value for U14 was also just below the threshold, but this snoRNA was previously shown to be affected by depletion of multiple individual helicases (Bohnsack et al., 2008). For snR55 (light gray in Figure 4), previous qPCR results suggested that this snoRNA was affected by depletion of several different helicases (Bohnsack et al., 2008). However, these apparent alterations could not be confirmed by northern blot analysis of the corresponding samples, and this is also the case for Prp43 depletion (data not shown).

The modification sites of snR39, snR39b, snR40, snR50, snR59, snR60, and snR72 cluster together, close to the major of Prp43 binding site at 25S+710 in helix 34 (Figure 2D), whereas snR70 targets sequences close to the Prp43-binding site at 18S helix 44.

**Figure 5:**
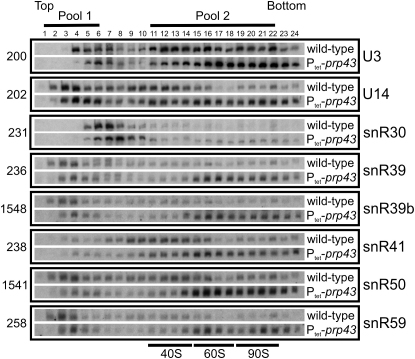
snoRNAs Are Retained on Preribosomes Following Depletion of Prp43 Strains were grown and samples prepared as in Figure 4. The distribution of a set of snoRNAs was analyzed by northern hybridization for each gradient fraction. The fractions pooled for Figures 4 and 6 as the free snoRNAs (pool 1) and preribosomes (pool 2) are indicated. Bars indicate fractions containing pre-40S, pre-60, and 90S preribosomes.

To substantiate the qPCR results, the degree of preribosome association of several affected snoRNAs and controls was assessed by northern hybridization of gradient fractions (Figure 5). RNA was extracted from sucrose density gradient fractions of cells grown in the presence (WT) or absence (P_tet_-*prp43*) of Prp43. The snoRNAs U3, snR30, U14, and snR41 were either unaltered or only modestly shifted to lower fractions. However, for snR39, snR39b, snR50, and snR59, the shift from the free form (pool 1) into the fractions containing preribosomal particles (pool 2) was more marked, reflecting increased accumulation of these snoRNAs in the preribosomal fractions, while the total levels of the snoRNAs remained unchanged (Figure S2). This indicates that depletion of Prp43 leads to accumulation of these snoRNAs in preribosomes.

### Mutations in DEAH Box Motifs I or III in Prp43 Block snoRNA Release

DEAD/H box RNA helicases are characterized by a conserved helicase domain composed of motifs I–VI (reviewed in Cordin et al., 2006; Bleichert and Baserga, 2007). Motif I represents a Walker A motif that is required for nucleotide binding, whereas motif III has been proposed to interlink the ATPase and helicase domains (Pause and Sonenberg, 1992; Plumpton et al., 1994). To determine whether the RNA helicase activity of Prp43 is required for snoRNA release, we made use of a point mutation in motif I (*prp43*-T123A), which was previously shown in vitro to block Prp43 ATPase activity, and a mutation in motif III (SAT domain; *prp43*-S247A) (Martin et al., 2002). WT and mutant forms of Prp43 were expressed from a centromeric plasmid in the P_tet_-*prp43* strain. Growth complementation was analyzed in liquid culture and on plates by spotting a 10-fold dilution series, for cells carrying plasmids with WT, *prp43*-T123A, or *prp43*-S247A genes, or the empty vector (Figure S3). Cells carrying the plasmid expressing WT Prp43 retained the WT growth rate after depletion of genomic-encoded Prp43 by doxycycline treatment. Expression of the Prp43 mutants from its own promoter did not result in the dominant-negative effects previously observed upon strong overexpression from a P_GAL_ promoter. Expression of Prp43*-*T123A gave the same growth defect as the empty plasmid indicating that the motif I mutation was lethal, as previously reported (Martin et al., 2002), whereas the Prp43*-*S247A mutant in motif III supported intermediate growth.

**Figure 6:**
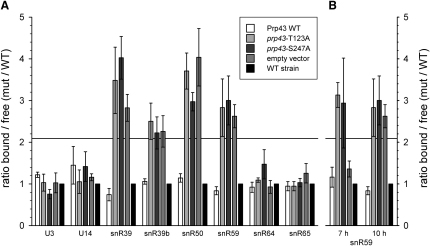
Analysis of snoRNA Distribution in Prp43 Mutant Strains The distribution of snoRNAs dependent on Prp43 for their release from preribosomes was analyzed in strains depleted of genomic *PRP43* but expressing WT or mutant Prp43 from a plasmid (see Figure S3). Samples were processed for qPCR as described in Figure 4, and averages of at least four independent experiments are shown. The line at 2.1 indicates the 95% confidence interval. Error bars represent standard error. (A) Depletion of genomic *PRP43* was performed for 10 hr in medium containing 20 μg/ml doxycycline. While U3, U14, snR64, and snR65 showed no effect, only expression of WT Prp43, but not the mutants or empty vector, could prevent accumulation of snR39, snR39b, snR50, and snR59 in fractions containing preribosomes. (B) Shown is comparison of effects on snR59 distribution after 7 or 10 hr of genomic *PRP43* depletion. After 7 hr depletion, cells expressing the Prp43 mutants T123A or S247A show a dominant-negative effect on snoRNA accumulation as compared to cells carrying the empty vector. After 10 hr depletion, cells carrying the empty vector showed similar effects as cells expressing one of the mutants, while expression of the WT protein resulted in the WT distribution of snR59.

The effects of the mutations in Prp43 on snoRNA release from preribosomes were tested by qPCR (Figure 6). The snoRNAs, U3, and snR65 showed no significant changes in any of the strains tested following doxycycline treatment for 10 hr in minimal medium. The distribution of snR64 also showed no clear differences. However, the rate of cell growth and ribosome synthesis in this experiment was lower than in the experiment shown in Figure 4, due to the use of selective minimal medium for plasmid analyses. A decreased growth rate can correlate with an increase in the free pools of snoRNAs (see, for example, Kos and Tollervey, 2005), perhaps favoring efficient pre-rRNA association in the absence of functional Prp43. In contrast, the snoRNAs snR39, snR39b, snR50, and snR59 showed preribosomal accumulation in Prp43-depleted cells (empty vector) and cells expressing the Prp43-T123A (domain I) or Prp43-S247A (domain III) mutants (Figure 6A).

Comparison of different depletion times (Figure 6B) showed that expression of either of the Prp43 mutants resulted in a dominant-negative phenotype after 7 hr depletion, since snoRNA accumulation in preribosomes was greater than for the Prp43-depleted, empty vector control. However, the phenotypes of the mutants were similar to Prp43 depletion after 10 hr.

Prp43 fulfills multiple functions during ribosome biogenesis and splicing. A possible explanation for the difference in strength of the effects caused by the mutations would be that there could be mechanistic differences in the various functions of Prp43. We therefore tested the levels of several pre-rRNAs and actin (*ACT1*) mRNA (as an example of a spliced mRNA) in the complementation strains after 10 hr of Prp43 depletion (Figure S4). *ACT1* mRNA levels were lowest in the strain carrying empty vector, indicating that the mutants might partially complement under these conditions, but little pre-mRNA accumulation was seen. The reduction in 27SB and accumulation of 35S was highest in the mutant strains, consistent with dominant-negative effects (Figure S4). The differences may reflect the participation of distinct Prp43 cofactors on its diverse substrates.

We conclude that ATP binding, and probably ATP hydrolysis by Prp43, is required for efficient snoRNA release from preribosomes.

## Discussion

To identify binding sites of Prp43 on RNA substrates, we used the CRAC technique, which allows the cloning and sequencing of short RNA fragments that correspond to the binding site(s) of a tagged protein (Granneman et al., 2009). Crosslinking was either performed in vivo before cell lysis or in vitro after enrichment of Prp43-containing complexes. Since Prp43 has been implicated in pre-mRNA splicing and pre-rRNA processing, we expected to find binding sites in both classes of RNA.

The spliceosomal snRNAs U2, U5, and U6 were previously reported to coprecipitate with Prp43 (Lebaron et al., 2005; Combs et al., 2006; Leeds et al., 2006), in line with the function of Prp43 in spliceosome disassembly. In our analyses, U6 was significantly crosslinked to Prp43 in vivo, whereas U2 and U5 were not (Figure 3). This suggests that Prp43 directly contacts U6, while U2 and U5 may associate indirectly with Prp43 as components of a postsplicing complex. No significant enrichment of intronic or exonic sequences was observed for Prp43 relative to the control (Table 1, Figure S1). It may be that interactions between Prp43 and the pre-mRNAs are very transient, or are lost during incubation of the cells on ice prior to and during UV irradiation. Prp43 interacts with the G-patch splicing factor Spp382 (Ntr1) and is a component of the NTR complex (Tsai et al., 2005, 2007; Boon et al., 2006; Tanaka et al., 2007). This suggests that Prp43 recruitment to the spliceosome might predominately be mediated by protein-protein interactions and U6, with only transient interactions to other RNA species during unwinding.

The large majority of sequences identified for Prp43 in the CRAC approach were mapped to rRNA (Table 1). These largely clustered around five putative Prp43-binding sites (Figure 2), and additional experimental data currently provide strong support for the functional significance of at least three of these sites. The differences in the Prp43 profiles found between in vivo and in vitro crosslinking experiments were unexpected, as previous analyses of the box C/D snoRNP proteins had not revealed similar differences (Granneman et al., 2009). This presumably reflects functional distinctions between snoRNPs and the helicase. Prior to and during in vivo crosslinking, the living cells are incubated on ice, whereas crosslinking in vitro is preceded by cell lysis, affinity purification on an IgG column, and elution by TEV cleavage, during which Prp43 remains potentially active. We speculate that different interactions are preferentially lost during these different preparations. It is therefore possible that, even though we detect multiple Prp43-binding sites, still further sites have been overlooked in both these analyses. Moreover, snoRNAs that were crosslinked to Prp43 are not all predicted to associate with preribosomes at sites close to the major Prp43 crosslinking sites, making likely that further Prp43-binding sites do indeed exist.

One putative binding site of Prp43 was located at the base of helix 44 in the 18S rRNA sequence, while the others were located in the 25S sequence (Figure 2). Prp43 was previously shown to coprecipitate with the 20S and 27S pre-rRNAs (Lebaron et al., 2005; Combs et al., 2006; Leeds et al., 2006), but not the mature rRNAs. This indicates that the helicase is recruited to these regions of the pre-rRNAs prior to final rRNA maturation. Prp43 and its cofactor Pfa1 interact genetically with Ltv1, a component of late pre-40S ribosomes, and with the endonuclease Nob1 that mediates cleavage of the 20S pre-rRNA at site D (Pertschy et al., 2009). Prp43 was therefore proposed to function in RNA unwinding or structural remodeling of the pre-40S particle, allowing access for the Nob1 endonuclease to site D. The Prp43-binding sites identified by in vivo CRAC at the 3′ end of the 18S rRNA in helix 44 lie close to site D (Figure 2A), strongly supporting this model.

Four putative binding sites were identified for Prp43 in the 25S sequence; in vivo CRAC revealed one prominent peak (between helices 39/40), whereas in vitro CRAC identified three major Prp43-binding sites (helices 23/24, 34, and 83) (Figure 1). The major in vivo binding site around 25S+1145 (helices 39/40) is close to modification sites directed by the box H/ACA snoRNA snR5 and the box C/D snR61 (Figure 2B). There are no data to support an interaction with snR5, but pre-rRNA association of snR61 showed some reduction after Prp43 depletion, and snR61 itself was crosslinked to Prp43 above background levels.

The Prp43-binding site at helices 23/24 lies within the region of 25S that base pairs to the 5.8S rRNA (Figure 2C). The functional significance of Prp43 binding here has yet to be assessed, but it is conceivable that it plays some role in the establishment of correct 25S-5.8S interactions.

Prp43 bound to 25S rRNA helix 34 appears to function in the release of snoRNAs from pre-60S ribosomal subunits. Six different box C/D snoRNAs, snR39, snR39b, snR50, snR59, snR60, and snR72, with sites of pre-rRNA base pairing close to the Prp43-binding site (see Figure 2D), were each trapped in association with preribosomes following depletion of Prp43 (Figures 4 and 5). A further box C/D species, snR40, showed a more minor effect, whereas two other snoRNAs that also bind within this general region, snR58 and snR80, were unaffected by Prp43 depletion. This indicates that snoRNA retention does not simply reflect some global defect in preribosome structure. More detailed analysis of this region revealed that rRNA sequences corresponding to the base pairing sites of several snoRNAs of this cluster were enriched with Prp43. These correspond to some of the smaller peaks visible in Figure 1, such as nucleotides 25S+792–810, which overlap with the base pairing sites of snR39/snR59 (Am807) and snR39b (Gm805), and nucleotides 25S+906–927 for snR60 (Gm908). Moreover, snR72, snR60, and, to a lesser extent, snR40 were directly crosslinked to Prp43. Why this particular subset of the snoRNAs in the cluster was crosslinked is currently unclear. The modification guide sequences of the snoRNAs were specifically enriched for Prp43 crosslinks, and nucleotide substitutions, which indicate the precise crosslinking site, were frequently found in or close to the guide sequences (Figure 3). These results support the direct unwinding of these pre-rRNA/snoRNA interactions by Prp43.

Efficient snoRNA release from preribosomes required the catalytic activity of Prp43, as shown by analysis of point mutations (Figure 6). The mutation in motif I was previously reported to strongly reduce the ATPase activity of Prp43 (Martin et al., 2002), whereas the motif III (SAT domain) mutant is predicted to uncouple ATPase and helicase activities (Pause and Sonenberg, 1992; Plumpton et al., 1994; Martin et al., 2002; Bernstein et al., 2006; Granneman et al., 2006). Expression of either protein failed to support normal release of snoRNAs when endogenous Prp43 was depleted.

We propose that the ATPase activity of Prp43 bound to helix 34 in the 27S pre-rRNA specifically mediates the unwinding from the rRNA of several box C/D snoRNAs that have binding sites located in close proximity within the preribosome.

The Prp43-binding site at helix 83 is also located close to a prominent cluster of snoRNA-directed modification sites. The dissociation of snoRNAs in this region was not clearly dependent on Prp43 activity. However, following Prp43 depletion, we observed reduced preribosome association for snR67, which modifies two sites flanking helix 83 (G2619 and U2724). A direct role for Prp43 in snoRNA interactions in the helix 83 region was shown by the identification of numerous crosslinks with the guide region of snR51, which directs modification close to helix 83 at U2729, overlapping an snR67-binding site. In an attempt to better understand these interactions, the profile of crosslinked rRNA sequences obtained for this region was analyzed in closer detail. Only few hits were found from the region immediately encompassing the modified nucleotides 2724 (snR67) and 2729 (snR51), whereas rRNA sequences from the other base pairing site of snR67 around G2619 were strongly enriched with Prp43 (nucleotides 2605–2625). This suggested that Prp43 bound to helix 83 allows access of snR67 to its base pairing site around G2619 in helix 80. However, Prp43 may be redundant with other factors for the access and release of snR51 and snR67 in helix 86.

Prp43 depletion was also associated with reduced preribosome association of snR64, and consistent with this, methylation of C2337 in 25S rRNA was previously reported to be defective in strains lacking Prp43 (Leeds et al., 2006).

Even within clusters in which snoRNA binding was affected, depletion or mutation of Prp43 did not affect the binding or release of all snoRNAs predicted to associate with the pre-rRNA in the vicinity of its binding sites. This may reflect a degree of functional redundancy between some of the 19 helicases implicated in ribosome synthesis. Alternatively, different combinations of snoRNAs and helicases may function together at distinct times during ribosome maturation. Within snoRNA clusters, overlapping, mutually exclusive sites of snoRNA binding on the pre-rRNA are common, and there is some evidence for a temporal order in snoRNA binding (Kos and Tollervey, 2005). It is also possible that at least some snoRNAs are able to bind and dissociate from the pre-rRNA without the aid of helicase activity.

DEAD/H box RNA helicases have been proposed to act nonprocessively in RNA strand displacement, within a radius of their binding site (see, for example, Yang et al., 2007). The apparent role of Prp43 in promoting the association and/or dissociation of multiple snoRNAs within an rRNA domain would strongly support such a model. To our knowledge, Prp43 is the first RNA helicase involved in eukaryotic ribosome biogenesis for which binding sites on the pre-rRNA have been identified—and a surprisingly complex picture has emerged. Prp43 makes multiple, probably transient and functionally distinct interactions with the preribosomes. Roles in pre-rRNA cleavage and snoRNA binding and release can be assigned to Prp43 proteins bound at distinct individual sites. Whether such striking complexity is common among RNA helicases and/or the many other known ribosome synthesis factors remains to be determined.

## Experimental Procedures

### Yeast Strains and Growth Complementation

For the CRAC approach, the genomic copy of *PRP43* (or *ROK1*) was C-terminally tagged (Longtine et al., 1998) for expression as a Prp43-His_6_-TEV-2ProteinA fusion in the BY4741 (MATa; his3Δ1; leu2Δ0; met15Δ0; ura3Δ0) background. For complementation analysis, WT Prp43 and the indicated mutants (including 465 nucleotides upstream and 446 nucleotides downstream of the open reading frame) were cloned into the pRS415 vector for expression from the own promoter. Oligonucleotides used for cloning are shown in Table S1. Plasmids were transformed into a BY4741 derivative (YMK119; Bohnsack et al., 2008) with the genomic copy of *PRP43* under control of the tet^off^ promoter (P_tet_-*prp43*). For complementation analysis, cells were grown on plates or in liquid culture containing minimal medium without leucine for plasmid selection, while depletions for Figures 4 and 5 were carried out in YPD medium. Depletion was induced by addition of 20 μgml^−1^ or 5 μgml^−1^ doxycycline for complementation (Figure 6) or standard depletions (Figures 4 and 5), respectively. For complementation analysis on plates, cells were grown to OD_600_ 0.2 under permissive conditions, doxycycline was added for depletion conditions, and cells were kept in exponential phase for an additional 4 hr before spotting.

### Crosslinking and Identification of Prp43-Binding Sites

The CRAC method was performed as previously described in detail (Granneman et al., 2009). Briefly, crosslinking was performed using a Stratalinker (Stratagene) either in vivo in living cells or in vitro after the first step of complex enrichment. Complexes were first purified on IgG Sepharose followed by elution with TEV protease and immobilization on Nickel-NTA for RNA trimming, dephosphorylation, phosphorylation, and linker ligation steps. Reverse transcription and PCR were carried out after Proteinase K digestion. PCR products were either cloned and sequenced by the Sanger method or directly submitted for Solexa sequencing. The resulting sequences were analyzed by BLAST against a yeast genomic database.

### RNA Preparation and Quantitative PCR

RNA was prepared from cells grown in depletion conditions as previously described (Bohnsack et al., 2008). Briefly, cells were harvested and lysed, and soluble material was loaded on 12 ml 10%–45% sucrose gradients and centrifuged for 16 hr at 23,500 rpm in a TST41.14 rotor. Fractions (500 μl) were collected, and RNA was extracted as described (Sambrook et al., 1989). cDNA was prepared for qPCR as described (Bohnsack et al., 2008). cDNA from fractions containing free (pool 1) or preribosome bound snoRNAs (pool 2) was pooled (as indicated in Figure 5) followed by quantitative RT PCR analyzing all yeast snoRNAs. Data analysis was performed as described (Bohnsack et al., 2008). Five experiments were performed for Figure 4. The value showing the highest deviation from the median was omitted, and averages of the remaining four values are shown in Figure 4. The lines at values 2.1 and 0.48 in Figure 4 represent the 95% confidence cutoff calculated from the distribution of the logarithmic values for all 75 snoRNAs.

### Northern Blotting

Northern transfer and hybridization were performed as described by Sambrook et al. (1989). Hybridization probes are shown in Table S1.

### Analysis of Solexa Data

This was performed using Novoalign 2.04 as described previously (Granneman et al., 2009). Cluster generation used Cluster 3.0 and Java Treeview.

## Figures and Tables

**Table 1:** Prp43 Crosslinks to Cellular RNA

Prp43 In Vivo Crosslinking		
	Hits	Percent total
Genes	157,016	16.11
Intronic genes	4,356	0.45
rDNA	726,570	74.55
Box C/D snoRNAs	27,562	2.83
Box H/ACA snoRNAs	3,543	0.36
snRNAs	31,268	3.21
tRNAs	23,496	2.41
Intronic tRNAs	810	0.08
Total number of mapped reads	974,621	

Prp43 In Vitro Crosslinking		

	Hits	Percent total
Genes	24,118	1.60
Intronic genes	624	0.04
rDNA	1,478,415	98.03
Box C/D snoRNAs	2,024	0.13
Box H/ACA snoRNAs	380	0.03
snRNAs	2,419	0.16
tRNAs	219	0.01
Intronic tRNAs	0	0.00
Total number of mapped reads	1,508,199	

Control In Vivo Crosslinking		

	Hits	Percent total
Genes	122,505	23.73
Intronic genes	2,489	0.48
rDNA	374,291	72.50
Box C/D snoRNAs	290	0.06
Box H/ACA snoRNAs	542	0.10
snRNAs	887	0.17
tRNAs	14,803	2.87
Intronic tRNAs	423	0.08
Total number of mapped reads	516,230	

Cells expressing Prp43-HTP or control cells were exposed to UV crosslinking in vivo or enriched Prp43-HTP containing complexes were crosslinked in vitro. Crosslinked RNAs were identified as described for Figure 1. Identified RNAs were sorted into several categories, and for each category the number of hits and the percentage of total hits of the sample are shown.

## Supplemental Data

Document S1. Four Figures, Two Tables, and Supplemental Sequence Alignments
