# Supplementary material for: Prp43 Bound at Different Sites on the Pre-rRNA Performs Distinct Functions in Ribosome Synthesis
Source: Mol Cell. 2009 Nov 25;36(4-24):583–92. doi: 10.1016/j.molcel.2009.09.039 (PMC2806949; doi:10.1016/j.molcel.2009.09.039)
Supplement: Document S1. Four Figures, Two Tables, and Supplemental Sequence Alignments [file mmc1.pdf]

Molecular Cell, Volume *36*

Supplemental Data

Prp43 Bound at Different Sites on the Pre-rRNA

Performs Distinct Functions in Ribosome Synthesis

Markus T. Bohnsack, Roman Martin, Sander Granneman,
Maike Ruprecht, Enrico Schleiff, and David Tollervey

Figure S1: Prp43 crosslinks to cellular RNA. (A) Cells expressing HTP-tagged Prp43 or Rok1, or no tagged protein as control, were UV irradiated, tagged Prp43 and Rok1 were enriched on IgG sepharose (IgG eluate) and crosslinked RNA was trimmed and radiolabeled (labeled RNA). Proteins were separated by SDS PAGE and visualized by western blot (upper and middle panel) and crosslinked, radiolabelled RNA by autoradiography (lower panel). Crosslinked RNAs were then ligated to linkers, followed by RT-PCR and Solexa deep sequencing (B-E). Crosslinking with cells expressing Prp43-HTP was either performed *in vivo* (B) or *in vitro* (C). Untagged control cells were crosslinked *in vivo* (D). Identified sequences were sorted into categories shown in (E).

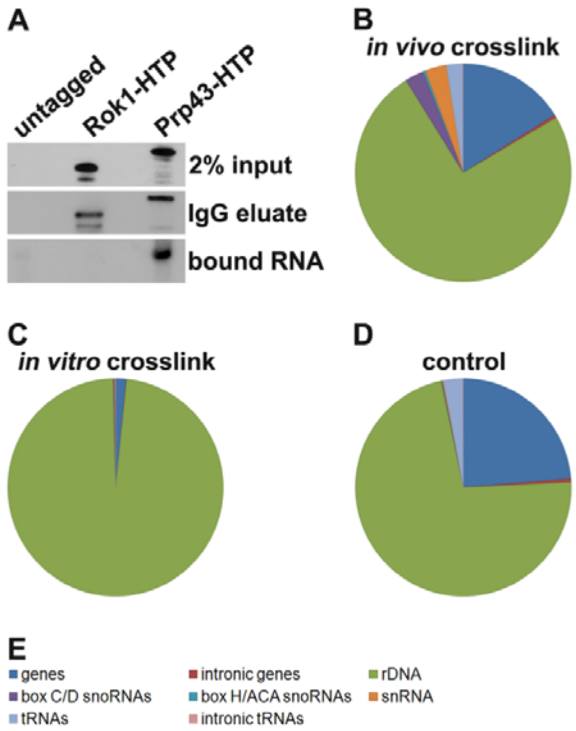

Figure S2: Analysis of snoRNA levels before and after depletion of Prp43.

Prp43 was depleted in the P_{tet}-*prp43* strain as described for Figure 4 and total RNA was isolated from cells followed by separation on a 10% acrylamide/8M urea gel and Northern transfer. snoRNAs were probed as indicated. No significant changes in the cellular levels of these snoRNAs were detected after Prp43 depletion.

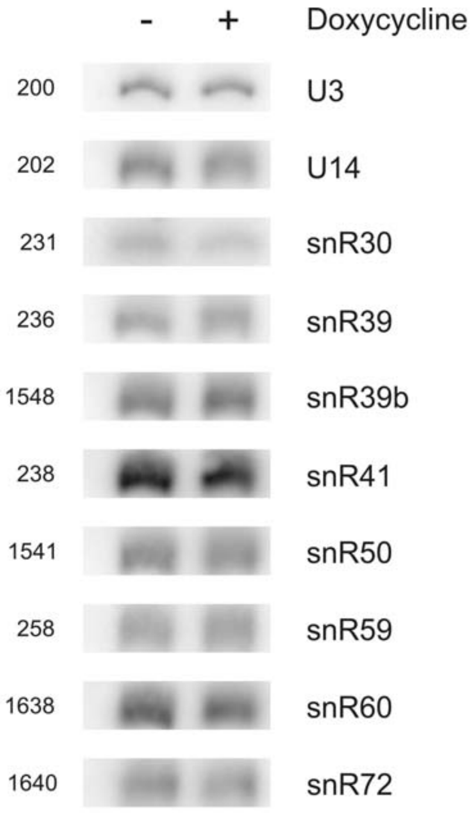

Figure S3: Analysis of Prp43 wild type and mutants for growth complementation.

A P_{tet}-*prp43* strain was transformed with plasmids expressing wild type Prp43 from its own promoter, the motif I mutant Prp43-T123A, motif III mutant Prp43-S247A, or with empty vector. (A, B) Growth was analyzed after spotting 10-fold dilutions of cultures on plates in the absence (permissive conditions; A) or presence (B) of doxycycline to repress chromosomal Prp43 expression. (C) Growth was followed in liquid culture for 24h after addition of doxycycline.

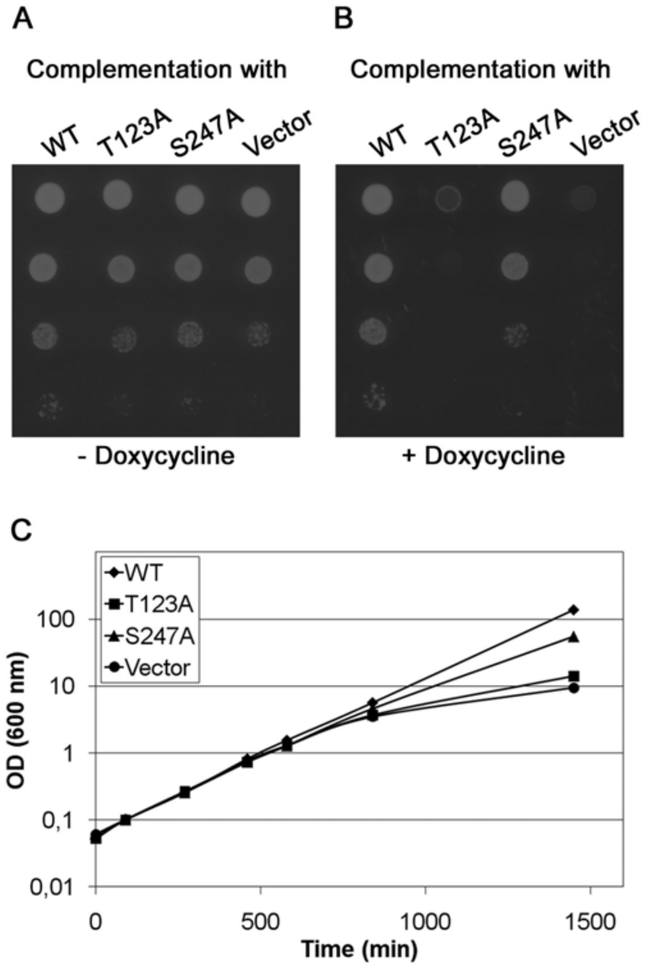

Figure S4: Northern blot analysis of pre-rRNA and actin mRNA levels in Prp43 mutant strains.

Genomic *PRP43* was depleted in cells expressing wildtype or mutant Prp43, or carrying empty vector. After 10 hours depletion cells were harvested, total RNA was isolated, separated on a 1.2% agarose gel followed by Northern transfer. The levels of several pre-ribosomal RNA intermediates and actin (*ACT1*) mRNA were probed as indicated (A). Levels of 27SB, 20S and actin mRNA were quantified (B). Expression of Prp43 mutants resulted in a slightly stronger reduction in pre-rRNA levels that in cells carrying the empty vector, whereas the opposite effect was observed on actin mRNA levels.

A

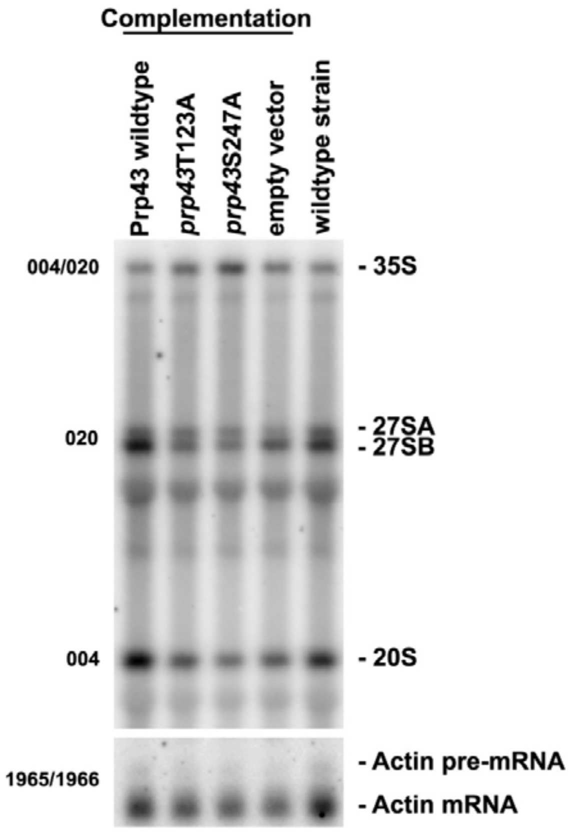

B

Quantification

95	50	33	78	84	27SB
128	77	67	89	109	20S
70	56	51	45	71	Actin mRNA

Table S1: Oligonucleotides used in this study.

Sequences of the oligonucleotides used as probes for Northern hybridisation or cloning of the *PRP43* gene including 465 nucleotides upstream and 446 nucleotides downstream of the open reading frame are shown.

No.	Sequence	Use
004	CGGTTTTAATTGTCCTA	pre-rRNA probe
020	TGAGAAGGAAATGACGCT	pre-rRNA probe
200	TTATGGGACTTGTT	U3a/b probe
202	TCACTCAGACATCCTAGG	U14 probe
231	CTAAGTTAAACTCGTCAACG	snR30 probe
236	GGTGATAAGTTACGACAGC	snR39 probe
238	GGGTTGTCGACATGTAGTTA	snR41 probe
258	GGTGATTAAACGACAGCATTGTCAAAGACTAGTCGA	snR59 probe
1396	ATATTACTCGAGATGCCAAGGAAGCGTACTGAGTACAG	FP Prp43 cloning
1397	ATTATTGCGGCCGCTGTAGTTCAAATTAGATAGTGG	RP Prp43 cloning
1541	GCTGCAAATTGCTACCTCTTTCA	snR50 probe
1548	TGCTAGTCACTTTTTGGAATGCC	snR39b probe
1638	CAATCAGTTGAACTATGCATCTTTGG	snR60 probe
1640	GATCAGACTGACGTGCAAATCATT	snR72 probe
1965	CAATAACCAAAGCAGCAAC	actin1 probe
1966	ATACCGGCAGATTCCAAACCC	actin1 probe

Table S2:

To generate the snoRNA hit table, we used a perl script for information from the novoalign output file (www.novocraft.com; version 2.04) to count the number of hits for each gene of interest from the total number of mapped reads.

Prp43 in vivo

----box CD snoRNAs----

snoRNA	hits	perc_hits
snR128	817	0.00084111
snR13	232	0.00023885
snR17a	715	0.0007361
snR17b	369	0.00037989
snR18	37	3.81E-05
snR190	267	0.00027488
snR24	406	0.00041798
snR38	62	6.38E-05
snR39	116	0.00011942
snR39B	136	0.00014001
snR4	219	0.00022546
snR40	664	0.0006836
snR41	45	4.63E-05
snR45	271	0.000279
snR47	403	0.00041489
snR48	0	0
snR50	83	8.54E-05
snR51	15582	0.01604186
snR52	663	0.00068257
snR53	13	1.34E-05
snR54	207	0.00021311
snR55	176	0.00018119
snR56	113	0.00011633
snR57	372	0.00038298
snR58	40	4.12E-05
snR59	250	0.00025738
snR60	1419	0.00146088
snR61	556	0.00057241
snR62	1	1.03E-06
snR63	282	0.00029032
snR64	383	0.0003943
snR65	0	0
snR66	18	1.85E-05
snR67	314	0.00032327
snR68	89	9.16E-05
snR69	155	0.00015957
snR70	130	0.00013384
snR71	143	0.00014722
snR72	2231	0.00229684
snR73	452	0.00046534
snR74	586	0.00060329
snR75	0	0
snR76	107	0.00011016
snR77	127	0.00013075
snR78	0	0
snR79	718	0.00073919
snR87	0	0

Control

----box CD snoRNAs----

snoRNA	hits	perc_hits
snR128	224	0.00047194
snR13	160	0.0003371
snR17a	0	0
snR17b	0	0
snR18	0	0
snR190	0	0
snR24	3	6.32E-06
snR38	0	0
snR39	0	0
snR39B	0	0
snR4	0	0
snR40	0	0
snR41	0	0
snR45	0	0
snR47	0	0
snR48	0	0
snR50	0	0
snR51	0	0
snR52	0	0
snR53	0	0
snR54	0	0
snR55	0	0
snR56	0	0
snR57	0	0
snR58	67	0.00014116
snR59	398	0.00083853
snR60	31	6.53E-05
snR61	0	0
snR62	0	0
snR63	0	0
snR64	0	0
snR65	0	0
snR66	0	0
snR67	0	0
snR68	0	0
snR69	0	0
snR70	0	0
snR71	0	0
snR72	0	0
snR73	0	0
snR74	0	0
snR75	0	0
snR76	0	0
snR77	0	0
snR78	0	0
snR79	0	0
snR87	0	0

Prp43 in vitro

----box CD snoRNAs----

snoRNA	hits	perc_hits
snR128	155	9.41E-05
snR13	10	6.07E-06
snR17a	254	0.00015415
snR17b	197	0.00011955
snR18	0	0
snR190	0	0
snR24	28	1.70E-05
snR38	0	0
snR39	0	0
snR39B	0	0
snR4	67	4.07E-05
snR40	47	2.85E-05
snR41	5	3.03E-06
snR45	9	5.46E-06
snR47	7	4.25E-06
snR48	0	0
snR50	0	0
snR51	90	5.46E-05
snR52	32	1.94E-05
snR53	19	1.15E-05
snR54	0	0
snR55	0	0
snR56	0	0
snR57	19	1.15E-05
snR58	15	9.10E-06
snR59	10	6.07E-06
snR60	18	1.09E-05
snR61	115	6.98E-05
snR62	3	1.82E-06
snR63	0	0
snR64	7	4.25E-06
snR65	0	0
snR66	0	0
snR67	36	2.18E-05
snR68	0	0
snR69	0	0
snR70	0	0
snR71	57	3.46E-05
snR72	198	0.00012016
snR73	73	4.43E-05
snR74	25	1.52E-05
snR75	0	0
snR76	232	0.0001408
snR77	0	0
snR78	0	0
snR79	14	8.50E-06
snR87	0	0

----box HACA snoRNAs----

snoRNA	hits	perc_hits
snR10	554	0.00057035
snR11	298	0.00030679
snR161	32	3.29E-05
snR189	10	1.03E-05
snR191	99	0.00010192
snR3	68	7.00E-05
snR30	990	0.00101922
snR31	135	0.00013898
snR33	494	0.00050858
snR34	0	0
snR35	196	0.00020178
snR36	11	1.13E-05
snR37	360	0.00037062
snR42	96	9.88E-05
snR43	2	2.06E-06
snR44	16	1.65E-05
snR46	0	0
snR49	3	3.09E-06
snR5	105	0.0001081
snR8	180	0.00018531
snR80	0	0
snR81	1	1.03E-06
snR82	234	0.00024091
snR83	453	0.00046637
snR84	40	4.12E-05
snR85	139	0.0001431
snR86	70	7.21E-05
snR9	26	2.68E-05

----box HACA snoRNAs----

snoRNA	hits	perc_hits
snR10	0	0
snR11	0	0
snR161	0	0
snR189	0	0
snR191	0	0
snR3	4	8.43E-06
snR30	498	0.00104922
snR31	0	0
snR33	22	4.64E-05
snR34	1	2.11E-06
snR35	0	0
snR36	0	0
snR37	0	0
snR42	0	0
snR43	0	0
snR44	0	0
snR46	0	0
snR49	0	0
snR5	0	0
snR8	1	2.11E-06
snR80	0	0
snR81	0	0
snR82	1	2.11E-06
snR83	86	0.00018119
snR84	0	0
snR85	0	0
snR86	0	0
snR9	0	0

----box HACA snoRNAs----

snoRNA	hits	perc_hits
snR10	245	0.00014868
snR11	0	0
snR161	0	0
snR189	0	0
snR191	0	0
snR3	1	6.07E-07
snR30	118	7.16E-05
snR31	0	0
snR33	0	0
snR34	0	0
snR35	91	5.52E-05
snR36	0	0
snR37	13	7.89E-06
snR42	0	0
snR43	0	0
snR44	0	0
snR46	0	0
snR49	0	0
snR5	23	1.40E-05
snR8	0	0
snR80	15	9.10E-06
snR81	0	0
snR82	0	0
snR83	26	1.58E-05
snR84	0	0
snR85	0	0
snR86	32	1.94E-05
snR9	38	2.31E-05

Bohnsack *et al*., legend to supplemental sequence alignments

Alignments were generated using a perl script that uses the information from the novoalign output file (www.novocraft.com; version 2.04) to mark the location of the deletions and substitutions in the reads and to align them to the reference sequence. Each alignment file contains a maximum of 200 nucleotide sequences which the program randomly selected from the novoalign output file. Substitutions in sequences are indicated in lower case, whereas deletions are indicated as gaps. Alignments of snoRNA hits for snR51, snR60 and snR72 and alignments with sequences around the 5 major peaks on the rDNA found after *in vivo* or *in vitro* cross-linking are shown.

snR51

```
                  1    .........|.........|.........|.........|.........|.........|.........|.........|.........|.........|....... 107
                     snR51     TATTATGATGATTTTTTTATATTCACACTGTACTAGATTGGTCTCTTTAACGAAGGGGCTAATTGATGACTACAAAATAAAAAATAACTGATTTAATGACTCTGAAA
@HWI-EAS293:2:1:1140:1582#0/1  ----------------------------------AGATTGGTCTCTTTAAC--------------------------------------------------------
@HWI-EAS293:2:1:445:2042#0/1   -------------------------------------------------------------------------AAAAT-AAAAATAACTGATTTAATGACTCTGAAA
@HWI-EAS293:2:1:724:476#0/1    -------------------------------------------------------------------------AAAATAAAA---AACTGATTTAATGACTCTGAAA
@HWI-EAS293:2:1:964:1308#0/1   -------------------------------------------------------------------------AAAAT-AAAAATAACTGATTTAATGACTCTGAAA
@HWI-EAS293:2:1:1576:59#0/1    -------------------------------------------------------------------------AAAATAAAAAATAACTGATTTAATGACTCTGAAA
@HWI-EAS293:2:1:1635:1118#0/1  -------------------------------------------------------------------------AAAATAAAAAATAACTGATTTAATGACTCTGAAA
@HWI-EAS293:2:1:1232:1807#0/1  --------------------------------------------------------------------------AAAT-AAAAATAACTGATTTAATGACTCTGAAg
@HWI-EAS293:2:1:162:726#0/1    ---------------------------------------------------------------------------AAaAtAAAAgAACTGATTTAATGACTCTaAA-
@HWI-EAS293:2:1:236:1407#0/1   ---------------------------------------------------------------------------AAaAtAAAATAACTGATTTAATGACTCTGAAA
@HWI-EAS293:2:1:519:1279#0/1   ---------------------------------------------------------------------------AAaAtAAAATAACTGATTTAATGACTCTGAAA
@HWI-EAS293:2:1:717:1121#0/1   ---------------------------------------------------------------------------AAaAtAAAATAACTGATTTAATGACTCTGAAA
@HWI-EAS293:2:1:796:1553#0/1   ---------------------------------------------------------------------------AAaAtAAAATAACTGATTTAATGACTCTGAAA
@HWI-EAS293:2:1:813:965#0/1    ---------------------------------------------------------------------------AAaAtAAAATAACTGATTTAATGACTCTGAAA
@HWI-EAS293:2:1:771:510#0/1    ----------------------------------------------------------------------------AaAAAAAATAACTGATTTAATGACT------
@HWI-EAS293:2:1:41:1797#0/1    ------------------------------------------------------------------------------AAAAAATAACTGATTTAATGACTCTGAAA
@HWI-EAS293:2:1:48:903#0/1     ------------------------------------------------------------------------------AAAAAATAACTGATTTAATGACTCTGTCA
@HWI-EAS293:2:1:99:1299#0/1    ------------------------------------------------------------------------------AAAAAATAACTGATTTAATGACTCTGAAA
@HWI-EAS293:2:1:100:989#0/1    ------------------------------------------------------------------------------AAAAAATAACTGATTTAATGACTCTGAAA
@HWI-EAS293:2:1:120:944#0/1    ------------------------------------------------------------------------------AAAAAATAACTGATTTAATGACTCTGAAA
@HWI-EAS293:2:1:127:205#0/1    ------------------------------------------------------------------------------AAAAAATAACTGATTTAATGACTCTGAAA
@HWI-EAS293:2:1:134:1771#0/1   ------------------------------------------------------------------------------AAAAAATAACTGATTTAATGACTCTGAAA
@HWI-EAS293:2:1:175:1835#0/1   ------------------------------------------------------------------------------AAAAAATAACTGATTTAATGACTCTGAAA
@HWI-EAS293:2:1:184:1463#0/1   ------------------------------------------------------------------------------AAAAAATAACTGATTTAATGAC-------
@HWI-EAS293:2:1:219:1733#0/1   ------------------------------------------------------------------------------AAAAAATAACTGATTTAATGAC-------
@HWI-EAS293:2:1:236:1551#0/1   ------------------------------------------------------------------------------AAAAAATAACTGATTTAATGACTCTGAAA
@HWI-EAS293:2:1:261:1951#0/1   ------------------------------------------------------------------------------AAAAAATAACTGATTTAATGACTCTGAAA
@HWI-EAS293:2:1:278:1994#0/1   ------------------------------------------------------------------------------AAAAAATAACTGATTTAATGACTCTGAAA
@HWI-EAS293:2:1:304:1641#0/1   ------------------------------------------------------------------------------AAAAAATAACTGATTTAATGACTCTGAAA
@HWI-EAS293:2:1:335:1759#0/1   ------------------------------------------------------------------------------AAAAAATAACTGATTTAATGACTCTGAAA
@HWI-EAS293:2:1:339:1565#0/1   ------------------------------------------------------------------------------AAAAAATcACTGATTTAATGACTCTGAAA
@HWI-EAS293:2:1:353:1392#0/1   ------------------------------------------------------------------------------AAAAAATAACTGATTTAATGACTCTGAAA
@HWI-EAS293:2:1:416:1805#0/1   ------------------------------------------------------------------------------AAAAAATAACTGATTTAATGACTCTGAAA
@HWI-EAS293:2:1:440:1373#0/1   ------------------------------------------------------------------------------AAAAAATAACTGATTTAATGACTCTGAAA
@HWI-EAS293:2:1:462:1239#0/1   ------------------------------------------------------------------------------AAAAAATAACTGATTTAATGACTCTGAAA
@HWI-EAS293:2:1:473:1874#0/1   ------------------------------------------------------------------------------AAAAAATAACTGATTTAATGACTCTGAAA
@HWI-EAS293:2:1:518:179#0/1    ------------------------------------------------------------------------------AAAAAATAACTGATTTAATGACTCTGAAA
@HWI-EAS293:2:1:526:1873#0/1   ------------------------------------------------------------------------------AAAAAATAACTGATTTAATGACTCTGAAA
@HWI-EAS293:2:1:536:527#0/1    ------------------------------------------------------------------------------AAAAAATAACTGATTTAATGACTCTGAAA
@HWI-EAS293:2:1:548:627#0/1    ------------------------------------------------------------------------------AAAAAATAACTGATTTAATGACTCTGAAA
@HWI-EAS293:2:1:550:1939#0/1   ------------------------------------------------------------------------------AAAAAATAACTGATTTAATGACTCTGAAA
@HWI-EAS293:2:1:577:408#0/1    ------------------------------------------------------------------------------AAAAAATAACTGATTTAATGACTCTGAAA
@HWI-EAS293:2:1:593:1518#0/1   ------------------------------------------------------------------------------AAAAAATAACTGATTTAATGcCTCTGAAA
@HWI-EAS293:2:1:637:896#0/1    ------------------------------------------------------------------------------AAAAAAcAACTGATTTAATGACTCTGAAA
@HWI-EAS293:2:1:647:1859#0/1   ------------------------------------------------------------------------------AAAAAATAACTGATTTAATGACTCTGAAA
@HWI-EAS293:2:1:647:1922#0/1   ------------------------------------------------------------------------------AAAAAATAACTGATTTAATGACTCTGAAA
@HWI-EAS293:2:1:654:14#0/1     ------------------------------------------------------------------------------AAAAAATAACTGATTTAATGACTCTGAAA
@HWI-EAS293:2:1:699:995#0/1    ------------------------------------------------------------------------------AAAAAATAACTGATTTAATGACTCTGAAA
@HWI-EAS293:2:1:766:561#0/1    ------------------------------------------------------------------------------AAAAAATAACTGATTTAATGACTCTGAAA
@HWI-EAS293:2:1:793:249#0/1    ------------------------------------------------------------------------------AAAAAATAACTGATTTAATGACTCTGAAA
@HWI-EAS293:2:1:815:1446#0/1   ------------------------------------------------------------------------------AAAAAATAACTGATTTAATGACTCTGAAA
@HWI-EAS293:2:1:839:178#0/1    ------------------------------------------------------------------------------AAAAAATAACTGATTTAATGACTCTGAAA
@HWI-EAS293:2:1:849:24#0/1     ------------------------------------------------------------------------------AAAAAATAACTGATTTAATGACTCTGAAA
@HWI-EAS293:2:1:855:693#0/1    ------------------------------------------------------------------------------AAAAAATAACTGATTTAATGACTCTGAAA
@HWI-EAS293:2:1:855:1751#0/1   ------------------------------------------------------------------------------AAAAAATAACTGATTTAATGACTCTGAAA
@HWI-EAS293:2:1:902:1681#0/1   ------------------------------------------------------------------------------AAAAAATAACTGATTTAATGACTCTGAAA
@HWI-EAS293:2:1:934:974#0/1    ------------------------------------------------------------------------------AAAAAATAACTGATTTAATGACTCTGAAA
@HWI-EAS293:2:1:1027:1875#0/1  ------------------------------------------------------------------------------AAAAAATAACTGATTTAATGACTCTGAAA
@HWI-EAS293:2:1:1053:1539#0/1  ------------------------------------------------------------------------------AAAAAATAACTGATTTAATGACTCTGAAA
@HWI-EAS293:2:1:1056:614#0/1   ------------------------------------------------------------------------------AAAAAATAACTGATTTAATGACTCTGAAA
@HWI-EAS293:2:1:1060:1069#0/1  ------------------------------------------------------------------------------AAAAAATAACTGATTTAATGACTCTGAAA
@HWI-EAS293:2:1:1065:1581#0/1  ------------------------------------------------------------------------------AAAAAAcAACTGATTTAATGACTCTGAAA
@HWI-EAS293:2:1:1104:1199#0/1  ------------------------------------------------------------------------------AAAAAATAACTGATTTAATGACTCTGAAA
@HWI-EAS293:2:1:1137:1980#0/1  ------------------------------------------------------------------------------AAAAAATAACTGATTTAATGACTCTGAAA
@HWI-EAS293:2:1:1156:1646#0/1  ------------------------------------------------------------------------------AAAAAATAACTGATTTAATGAC-------
@HWI-EAS293:2:1:1159:890#0/1   ------------------------------------------------------------------------------AAAAAATAACTGATTTAATGACTCTGAAA
@HWI-EAS293:2:1:1166:473#0/1   ------------------------------------------------------------------------------AAAAAATAACTGATTTAATGACTCTGAAA
@HWI-EAS293:2:1:1191:1823#0/1  ------------------------------------------------------------------------------AAAAAATAACTGATTTAATGACTCTGAAA
@HWI-EAS293:2:1:1195:786#0/1   ------------------------------------------------------------------------------AAAAAATAACTGATTTAATGACTCTGAAA
@HWI-EAS293:2:1:1224:1339#0/1  ------------------------------------------------------------------------------gAAAAATAACTGATTTAATGACTCTGAAA
@HWI-EAS293:2:1:1233:1041#0/1  ------------------------------------------------------------------------------AAAAAATAACTGATTTAATGACTCTGAAA
@HWI-EAS293:2:1:1252:1978#0/1  ------------------------------------------------------------------------------AAAAAATAACTGATTTAATGACTCTGAAA
@HWI-EAS293:2:1:1264:1417#0/1  ------------------------------------------------------------------------------AAAAAATAACTGATTTAATGACTCTGAAA
@HWI-EAS293:2:1:1266:58#0/1    ------------------------------------------------------------------------------AAAAAATAACTGATTTAATGACTCTGAA-
@HWI-EAS293:2:1:1268:1425#0/1  ------------------------------------------------------------------------------AAAAAATAACTGATTTAATGACTCTGAAA
@HWI-EAS293:2:1:1272:1353#0/1  ------------------------------------------------------------------------------AAAAAATAACTGATTTAATGACTCcGAAA
@HWI-EAS293:2:1:1278:471#0/1   ------------------------------------------------------------------------------AAAAAATAACTGATTTAATGACTCTGAAA
@HWI-EAS293:2:1:1311:1184#0/1  ------------------------------------------------------------------------------AAAAAATAACTGATTTAATGACTCTGAAA
@HWI-EAS293:2:1:1314:1285#0/1  ------------------------------------------------------------------------------AAAAAATAACTGATTTAATGACTCTGAAA
@HWI-EAS293:2:1:1321:1782#0/1  ------------------------------------------------------------------------------AAAAAATAACTGATTTAATGACTCTGAAA
@HWI-EAS293:2:1:1336:449#0/1   ------------------------------------------------------------------------------AAAAAATAACTGATTTAATGACTCTGAAA
@HWI-EAS293:2:1:1338:1365#0/1  ------------------------------------------------------------------------------AAAAAATAACTGATTTAATGACTCTGAAA
@HWI-EAS293:2:1:1346:834#0/1   ------------------------------------------------------------------------------AAAgAATAACTGATTTAATGACTCTGAAA
@HWI-EAS293:2:1:1401:1633#0/1  ------------------------------------------------------------------------------AAAAAATAACTGATTTAATGACTCTGAAA
@HWI-EAS293:2:1:1451:1749#0/1  ------------------------------------------------------------------------------AAAAAAcAACTGATTTAATGACTCTGAAA
@HWI-EAS293:2:1:1452:588#0/1   ------------------------------------------------------------------------------AAAAAAcAACTGATTTAATGACTCTGAAA
@HWI-EAS293:2:1:1478:833#0/1   ------------------------------------------------------------------------------AAAAAATAACTGATTTAATGACTCTGAAA
@HWI-EAS293:2:1:1492:1439#0/1  ------------------------------------------------------------------------------AAAAAATAACTGATTTAATGACTCTGAAA
@HWI-EAS293:2:1:1492:838#0/1   ------------------------------------------------------------------------------AAAAAATAACTGATTTAATGACTCTGAAA
@HWI-EAS293:2:1:1493:475#0/1   ------------------------------------------------------------------------------AAAAAATAACTGATTTAATGACTCTGAAA
@HWI-EAS293:2:1:1499:54#0/1    ------------------------------------------------------------------------------AAAAAATAACTGATTTAATGACTCTGAAA
@HWI-EAS293:2:1:1583:1301#0/1  ------------------------------------------------------------------------------AAAAAATAACTGATTTAATGACTCTGAAA
@HWI-EAS293:2:1:1585:669#0/1   ------------------------------------------------------------------------------AAAAAATAACTGATTTAATGACTCTGAAA
@HWI-EAS293:2:1:1594:1192#0/1  ------------------------------------------------------------------------------AAAAgAcAACTGATTTAATGACTCTGAAA
@HWI-EAS293:2:1:1594:226#0/1   ------------------------------------------------------------------------------AAAAAATAACTGATTTAATGACTCTGAAA
@HWI-EAS293:2:1:1601:967#0/1   ------------------------------------------------------------------------------AAAAAATAACTGATTTAATGACTCTGAAA
@HWI-EAS293:2:1:1645:571#0/1   ------------------------------------------------------------------------------AAAAAATAACTGATTTAATGACTCTGAAA
@HWI-EAS293:2:1:1645:913#0/1   ------------------------------------------------------------------------------AAAAAATAACTGATTTAATGACTCTGAAA
@HWI-EAS293:2:1:1660:1546#0/1  ------------------------------------------------------------------------------AAAAAAcAACTGATTTAATGACTCTGAAA
@HWI-EAS293:2:1:327:1899#0/1   -------------------------------------------------------------------------------AAAAATAACTGATTTAATGACTCTGAAA
@HWI-EAS293:2:1:367:1542#0/1   -------------------------------------------------------------------------------AAAAATAACTGATTTAATGACTCTGAAA
@HWI-EAS293:2:1:482:892#0/1    -------------------------------------------------------------------------------AAAAATAACTGATTTAATGACTCTGAAA
@HWI-EAS293:2:1:548:1150#0/1   -------------------------------------------------------------------------------AAAAATAACTGATTTAATGACTCTGAAA
@HWI-EAS293:2:1:719:714#0/1    -------------------------------------------------------------------------------AAAAATAACTGATTTAATGACTCTGAAA
@HWI-EAS293:2:1:766:415#0/1    -------------------------------------------------------------------------------AAAAATAACTGATTTAATGACTCTGAAA
@HWI-EAS293:2:1:874:1271#0/1   -------------------------------------------------------------------------------AAAAATAACTGATTTAATGACTCTGAAA
@HWI-EAS293:2:1:963:1403#0/1   -------------------------------------------------------------------------------AAAAATAACTGATTTAATGACTCTGAAA
@HWI-EAS293:2:1:1017:507#0/1   -------------------------------------------------------------------------------AAAAATAACTGATTTAATGACTCTGAAA
@HWI-EAS293:2:1:1127:1263#0/1  -------------------------------------------------------------------------------AAAAATAACTGATTTAATGAC-------
@HWI-EAS293:2:1:1157:1242#0/1  -------------------------------------------------------------------------------AAAAATAACTGATTTAATGACTCTGAAA
@HWI-EAS293:2:1:1276:1896#0/1  -------------------------------------------------------------------------------AAAAATAACTGATTTAATGACTCTGAAA
@HWI-EAS293:2:1:1322:1874#0/1  -------------------------------------------------------------------------------AAAAATAACTGATTTAATGACTCTGAAA
@HWI-EAS293:2:1:1367:941#0/1   -------------------------------------------------------------------------------AAAAATAACTGATTTAATGACTCTGAAA
@HWI-EAS293:2:1:1404:1119#0/1  -------------------------------------------------------------------------------AAAAATAACTGATTTAATGACTCTGAAA
@HWI-EAS293:2:1:1416:1114#0/1  -------------------------------------------------------------------------------AAAAATAACTGATTTAATGACTCTGAAA
@HWI-EAS293:2:1:1434:1374#0/1  -------------------------------------------------------------------------------AAAAATAACTGATTTAATGACTCTGAAA
@HWI-EAS293:2:1:1567:295#0/1   -------------------------------------------------------------------------------AAAAATAACTGATTTAATGACTCTGAAA
@HWI-EAS293:2:1:1694:1871#0/1  -------------------------------------------------------------------------------AAAAATAACTGATTTAATGACTCTGAAA
@HWI-EAS293:2:1:35:1019#0/1    --------------------------------------------------------------------------------AAAATAACTGATTTAATGACTCTGAAA
@HWI-EAS293:2:1:76:962#0/1     --------------------------------------------------------------------------------AAAAaAACTGATTTAATGACTCTGAAA
@HWI-EAS293:2:1:141:407#0/1    --------------------------------------------------------------------------------AAAATAACTGATTTAATGACTCTGAAA
@HWI-EAS293:2:1:169:1017#0/1   --------------------------------------------------------------------------------AAAATAACTGATTTAATGACTCTGAAA
@HWI-EAS293:2:1:266:485#0/1    --------------------------------------------------------------------------------AAAAaAACTGATcTAATGACTCTGAAA
@HWI-EAS293:2:1:274:1314#0/1   --------------------------------------------------------------------------------AAAATAACTGATTTAATGACTCTGAAA
@HWI-EAS293:2:1:282:450#0/1    --------------------------------------------------------------------------------AAAATAACTGATTTAATGACTCTGAAA
@HWI-EAS293:2:1:342:565#0/1    --------------------------------------------------------------------------------AAAAaAACTGATTTAATGACTCTGAAA
@HWI-EAS293:2:1:362:1297#0/1   --------------------------------------------------------------------------------AAAATAACTGATTTAATGACTCTGAAA
@HWI-EAS293:2:1:411:386#0/1    --------------------------------------------------------------------------------AAAATAACTGATTTAATGACcCTGAAA
@HWI-EAS293:2:1:506:1447#0/1   --------------------------------------------------------------------------------AAAATAACTGATTTAATGACTCTGAAA
@HWI-EAS293:2:1:517:283#0/1    --------------------------------------------------------------------------------AAAAaAACTGATTTAATGACTCTGAAA
```

```
@HWI-EAS293:2:1:533:661#0/1      --------------------------------------------------------------------------------AAAAaAACTGATTTAATGACTCTGAAA
@HWI-EAS293:2:1:610:1907#0/1     --------------------------------------------------------------------------------AAAATAACTGATTTAATGACTCTGAAA
@HWI-EAS293:2:1:614:2008#0/1     --------------------------------------------------------------------------------AAAATAACTGATTTAATGACTCTGAAA
@HWI-EAS293:2:1:633:462#0/1      --------------------------------------------------------------------------------AAAAaAACTGATTTAATGACTCTGAAA
@HWI-EAS293:2:1:647:1609#0/1     --------------------------------------------------------------------------------AAAATAACTGATTTAcTGACTCTGAAA
@HWI-EAS293:2:1:658:305#0/1      --------------------------------------------------------------------------------AAAATAACTGATTTAATGACTCTGAAA
@HWI-EAS293:2:1:722:1174#0/1     --------------------------------------------------------------------------------AAAATAACTGATTTAATGACTCTGAAA
@HWI-EAS293:2:1:754:71#0/1       --------------------------------------------------------------------------------AAAAaAACTGATTTAATGACTCTGAAA
@HWI-EAS293:2:1:793:623#0/1      --------------------------------------------------------------------------------AAAATAACTGATTTAATGACTCTGAAA
@HWI-EAS293:2:1:837:867#0/1      --------------------------------------------------------------------------------AAAAcAACTGATTTAATGACTCTGAAA
@HWI-EAS293:2:1:856:1712#0/1     --------------------------------------------------------------------------------AAAAaAACTGATTTAATGACTCTGAAA
@HWI-EAS293:2:1:888:637#0/1      --------------------------------------------------------------------------------AAAATAACTGATTTAATGACTCTGAAA
@HWI-EAS293:2:1:893:1910#0/1     --------------------------------------------------------------------------------AAAATAACTGATTTAATGACTCTGAAA
@HWI-EAS293:2:1:915:310#0/1      --------------------------------------------------------------------------------AAAATAACTGATTTAATGACTCTGAAA
@HWI-EAS293:2:1:932:287#0/1      --------------------------------------------------------------------------------AAAAaAACTGATTTAATGACTCTGAAA
@HWI-EAS293:2:1:977:1389#0/1     --------------------------------------------------------------------------------AAAATAACTGATTTAATGACTCTGAAA
@HWI-EAS293:2:1:1043:400#0/1     --------------------------------------------------------------------------------AAAAaAACTGATTTAATGACTCTGAAA
@HWI-EAS293:2:1:1060:539#0/1     --------------------------------------------------------------------------------AAAATAACTGATTTAATGAC-------
@HWI-EAS293:2:1:1077:1148#0/1    --------------------------------------------------------------------------------AAAAaAACTGATTTAATGACTCTGAAA
@HWI-EAS293:2:1:1083:657#0/1     --------------------------------------------------------------------------------AAAATAACTGATTTAATGACTCTGAAA
@HWI-EAS293:2:1:1088:458#0/1     --------------------------------------------------------------------------------AAAATAACTGATTTAATGACTCTGAAA
@HWI-EAS293:2:1:1112:889#0/1     --------------------------------------------------------------------------------AAAATAACTGATTTAATGACTCTGAAA
@HWI-EAS293:2:1:1117:151#0/1     --------------------------------------------------------------------------------AAAATAACTGATTTAATGACTCTGAAA
@HWI-EAS293:2:1:1134:697#0/1     --------------------------------------------------------------------------------AAAATAACTGATTTAATGACTCTGAAA
@HWI-EAS293:2:1:1178:1194#0/1    --------------------------------------------------------------------------------AAAAaAACTGATTTAATGACTCTGAAA
@HWI-EAS293:2:1:1278:1381#0/1    --------------------------------------------------------------------------------AAAATAACTGATTTAATGACTCTGAAA
@HWI-EAS293:2:1:1280:345#0/1     --------------------------------------------------------------------------------AAAATAACTGATTTAATGACTCTGAAA
@HWI-EAS293:2:1:1317:1179#0/1    --------------------------------------------------------------------------------AAAATAACTGATTTAATGACTCTGAAA
@HWI-EAS293:2:1:1335:515#0/1     --------------------------------------------------------------------------------AAAATAACTGATTTAATGACTCTGAAA
@HWI-EAS293:2:1:1366:1352#0/1    --------------------------------------------------------------------------------AAAAaAACTGATTTAATGACTCTGAAA
@HWI-EAS293:2:1:1392:1487#0/1    --------------------------------------------------------------------------------AAAAaAACTGATTTAATGACTCTGAcA
@HWI-EAS293:2:1:1400:1320#0/1    --------------------------------------------------------------------------------AAAATAACTGATTTAATGACTCTGAAA
@HWI-EAS293:2:1:1447:1848#0/1    --------------------------------------------------------------------------------AAAATAACTGATTTAATGACTCTGAAA
@HWI-EAS293:2:1:1464:1816#0/1    --------------------------------------------------------------------------------AAAATAACTGATTTAATGACTCTGAAA
@HWI-EAS293:2:1:1466:1506#0/1    --------------------------------------------------------------------------------AAAATAACTGATTTAATGACTCTGAAA
@HWI-EAS293:2:1:1516:1152#0/1    --------------------------------------------------------------------------------AAAAaAACTGATTTAATGACTCTGAAA
@HWI-EAS293:2:1:1579:603#0/1     --------------------------------------------------------------------------------AAAAgAACgGATTTAATGACTCTGAAA
@HWI-EAS293:2:1:1601:386#0/1     --------------------------------------------------------------------------------AAAATAACTGATTTAATGA--CTGAAA
@HWI-EAS293:2:1:1671:964#0/1     --------------------------------------------------------------------------------AAAATAACTGATTTAATGACTCTGAAA
@HWI-EAS293:2:1:1705:260#0/1     --------------------------------------------------------------------------------AAAATAACTGATTTAATGACTCTGAAA
@HWI-EAS293:2:1:18:696#0/1       ---------------------------------------------------------------------------------AAAaAACTGATTTAATGACTCTGAAA
@HWI-EAS293:2:1:235:957#0/1      ---------------------------------------------------------------------------------AAAaAACTGATTTAATGACTCTGAAA
@HWI-EAS293:2:1:352:1153#0/1     ---------------------------------------------------------------------------------AAATAACTGATTTAATGACTCTGAcA
@HWI-EAS293:2:1:399:1473#0/1     ---------------------------------------------------------------------------------AAAaAACTGATTTAATGACTCTGAAA
@HWI-EAS293:2:1:400:1615#0/1     ---------------------------------------------------------------------------------AAAaAACTGATTTAATGACTCTGAAA
@HWI-EAS293:2:1:569:1682#0/1     ---------------------------------------------------------------------------------AAAaAACTGATTTAATGACTCTGAAA
@HWI-EAS293:2:1:627:375#0/1      ---------------------------------------------------------------------------------AAAaAACTGATTTAATGACTCTGAAA
@HWI-EAS293:2:1:690:1384#0/1     ---------------------------------------------------------------------------------AAAaAACTGATTTAATGACcCTGAAA
@HWI-EAS293:2:1:699:158#0/1      ---------------------------------------------------------------------------------AAATAACTGATTTAATGACTCTGAAA
@HWI-EAS293:2:1:705:1173#0/1     ---------------------------------------------------------------------------------AAAaAACTGATTTAATGACTCTGAAA
@HWI-EAS293:2:1:808:311#0/1      ---------------------------------------------------------------------------------AAAaAACTGATTTAATGACTCTGAAA
@HWI-EAS293:2:1:976:1266#0/1     ---------------------------------------------------------------------------------AAAaAACTGATTTAATGACTCTGAAA
@HWI-EAS293:2:1:1034:379#0/1     ---------------------------------------------------------------------------------AAATAACTGATTTAATGACTCTGAAA
@HWI-EAS293:2:1:1077:358#0/1     ---------------------------------------------------------------------------------AAAaAACTGATTTAATGACTCTGAAA
@HWI-EAS293:2:1:1103:184#0/1     ---------------------------------------------------------------------------------AAAaAACTGATTTAATGACTCTGAAA
@HWI-EAS293:2:1:1209:542#0/1     ---------------------------------------------------------------------------------AAAaAACTGATTTAATGACTCTGAAA
@HWI-EAS293:2:1:1275:987#0/1     ---------------------------------------------------------------------------------AAATAACTGATTTAATGACTCTGAAA
@HWI-EAS293:2:1:1328:1541#0/1    ---------------------------------------------------------------------------------AAAaAACTGATTTAATGACTCTGAAA
@HWI-EAS293:2:1:1385:1670#0/1    ---------------------------------------------------------------------------------AAAaAACTGATTTAATGACTCTGAAg
@HWI-EAS293:2:1:1465:1258#0/1    ---------------------------------------------------------------------------------AAAaAACTGATTTAATGACTCTGAAA
@HWI-EAS293:2:1:1483:1878#0/1    ---------------------------------------------------------------------------------AAAaAACTGATTTAATGACTCTGAAA
@HWI-EAS293:2:1:1560:1802#0/1    ---------------------------------------------------------------------------------AAAaAACTGATTTAATGACTCTGAAA
@HWI-EAS293:2:1:1610:851#0/1     ---------------------------------------------------------------------------------AAAaAACTGATTTAATGACTCTGAAA
@HWI-EAS293:2:1:1669:444#0/1     ---------------------------------------------------------------------------------AAAaAACTGATTTAATGACTCTGAAA
@HWI-EAS293:2:1:1691:906#0/1     ---------------------------------------------------------------------------------AAAaAACTGATTTAATGACTCTGAAA
@HWI-EAS293:2:1:1700:1522#0/1    ---------------------------------------------------------------------------------AAATAACTGATTTAATGACTCTGAAA
@HWI-EAS293:2:1:1711:1875#0/1    ---------------------------------------------------------------------------------AAAaAACTGATTTAATGACTCTGAAA
@HWI-EAS293:2:1:196:316#0/1      ------------------------------------------------------------------------------------aAACTGATTTAATGACT------
@HWI-EAS293:2:1:1010:1300#0/1    ------------------------------------------------------------------------------------aAACTGATTTAATGACT------
@HWI-EAS293:2:1:159:66#0/1       -------------------------------------------------------------------------------------AACTGATTTAATGACTCTGAAA
@HWI-EAS293:2:1:1013:1838#0/1    -------------------------------------------------------------------------------------AACTGATTTAATGACTCTGAgA
@HWI-EAS293:2:1:1099:1047#0/1    -------------------------------------------------------------------------------------AACTGATTTAATGACTCTGAAA
```

snR60

```
                              1    .........|.........|.........|.........|.........|.........|.........|.........|.........|.........|.... 104
                          snR60    TGTTAATGATGATAACCAAAGATGCATAGTTCAACTGATTGAACATACTATCGAAATGAAGATAAAAATTTCCATCGAAATTAGTCTTTCGCTCCTATCTGAAC
    @HWI-EAS293:2:1:870:33#0/1    -----------------AAAGATGC--AGTTCAACTGATTGAAC------------------------------------------------------------
  @HWI-EAS293:2:1:1762:1146#0/1    -----------------AAAGATGC--AGTTCAACTGATTGAAC------------------------------------------------------------
   @HWI-EAS293:2:3:1733:181#0/1    -----------------AAAGATGC--AGTTCAACTGATTGAAC------------------------------------------------------------
   @HWI-EAS293:2:5:1433:386#0/1    -----------------AAAGATGC--AGTTCAACTGATTGAAC------------------------------------------------------------
      @HWI-EAS293:2:8:8:527#0/1    -----------------AAAGATGC--AGTTCAACTGATTGAAC------------------------------------------------------------
   @HWI-EAS293:2:8:629:1597#0/1    -----------------AAAGATGC--AGTTCAACTGATTGAAC------------------------------------------------------------
  @HWI-EAS293:2:8:1049:1753#0/1    -----------------AAAGATGC--AGTTCAACTGATTGAAC------------------------------------------------------------
  @HWI-EAS293:2:8:1078:1508#0/1    -----------------AAAGATGC--AGTTCAACTGATTGAAC------------------------------------------------------------
   @HWI-EAS293:2:11:397:772#0/1    -----------------AAAGATGC--AGTTCAACTGATTGAAC------------------------------------------------------------
  @HWI-EAS293:2:12:297:1936#0/1    -----------------AAAGATGC--AGTTCAACTGATTGAAC------------------------------------------------------------
   @HWI-EAS293:2:14:273:365#0/1    -----------------AAAGATGC--AGTTCAACTGATTGAAC------------------------------------------------------------
     @HWI-EAS293:2:1:58:474#0/1    -------------------------ATAGTTCAACTGATTGAAC------------------------------------------------------------
   @HWI-EAS293:2:1:641:1937#0/1    -------------------------ATAGTTCAACTGATTGAAC------------------------------------------------------------
  @HWI-EAS293:2:1:1707:1314#0/1    -------------------------ATAGTTCAACTGATTGAAC------------------------------------------------------------
   @HWI-EAS293:2:2:430:1679#0/1    -------------------------ATAGTTCAACTGATTGAAC------------------------------------------------------------
  @HWI-EAS293:2:2:1027:1362#0/1    -------------------------ATAGTTCAACTGATTGAAC------------------------------------------------------------
  @HWI-EAS293:2:2:1051:1626#0/1    -------------------------ATAGTTCAACTGATTGAAC------------------------------------------------------------
   @HWI-EAS293:2:2:1312:726#0/1    -------------------------ATAGTTCAACTGATTGAAC------------------------------------------------------------
   @HWI-EAS293:2:2:1652:123#0/1    -------------------------ATAGTTCAACTGATTGAAC------------------------------------------------------------
   @HWI-EAS293:2:2:1683:170#0/1    -------------------------ATAGTTCAACTGATTGAAC------------------------------------------------------------
   @HWI-EAS293:2:3:336:1785#0/1    -------------------------ATAGTTCAACTGATTGAAC------------------------------------------------------------
    @HWI-EAS293:2:3:512:137#0/1    -------------------------ATAGTTCAACTGATTGAAC------------------------------------------------------------
    @HWI-EAS293:2:3:673:643#0/1    -------------------------ATAGTTCAACTGATTGAAC------------------------------------------------------------
  @HWI-EAS293:2:3:1098:1096#0/1    -------------------------ATAGTTCAACTGATTGAAC------------------------------------------------------------
    @HWI-EAS293:2:4:270:989#0/1    -------------------------ATAGTTCAACTGATTGAAC------------------------------------------------------------
   @HWI-EAS293:2:5:1698:789#0/1    -------------------------ATAGTTCAACTGATTGAAC------------------------------------------------------------
   @HWI-EAS293:2:6:387:1141#0/1    -------------------------ATAGTTCAACTGATTGAAC------------------------------------------------------------
   @HWI-EAS293:2:7:360:1604#0/1    -------------------------ATAGTTCAACTGATTGAAC------------------------------------------------------------
   @HWI-EAS293:2:7:491:1233#0/1    -------------------------ATAGTTCAACTGATTGAAC------------------------------------------------------------
    @HWI-EAS293:2:7:795:307#0/1    -------------------------ATAGTTCAACTGATTGAAC------------------------------------------------------------
   @HWI-EAS293:2:8:220:1330#0/1    -------------------------ATAGTTCAACTGATTGAAC------------------------------------------------------------
   @HWI-EAS293:2:8:1207:805#0/1    -------------------------ATAGTTCAACTGATTGAAC------------------------------------------------------------
    @HWI-EAS293:2:9:928:513#0/1    -------------------------ATAGTTCAACTGATTGAAC------------------------------------------------------------
   @HWI-EAS293:2:11:957:600#0/1    -------------------------ATAGTTCAACTGATTGAAC------------------------------------------------------------
  @HWI-EAS293:2:12:1175:974#0/1    -------------------------ATAGTTCAACTGATTGAAC------------------------------------------------------------
  @HWI-EAS293:2:13:677:2021#0/1    -------------------------ATAGTTCAACTGATTGAAC------------------------------------------------------------
 @HWI-EAS293:2:13:1451:1811#0/1    -------------------------ATAGTTCAACTGATTGAAC------------------------------------------------------------
 @HWI-EAS293:2:13:1744:1729#0/1    -------------------------ATAGTTCAACTGATTGAAC------------------------------------------------------------
  @HWI-EAS293:2:14:349:1778#0/1    -------------------------ATAGTTCAACTGATTGAAC------------------------------------------------------------
    @HWI-EAS293:2:1:589:790#0/1    ---------------------------AGTTCAACTGATTGAAC------------------------------------------------------------
   @HWI-EAS293:2:1:733:2024#0/1    ---------------------------AGcTCAACTGATTGAAC------------------------------------------------------------
  @HWI-EAS293:2:1:1153:1256#0/1    ---------------------------AGTTCAACTGATTGAAC------------------------------------------------------------
  @HWI-EAS293:2:1:1332:1257#0/1    ---------------------------AGcTCAACTGATTGAAC------------------------------------------------------------
    @HWI-EAS293:2:2:566:709#0/1    ---------------------------AGcTCAACTGATTGAAC------------------------------------------------------------
    @HWI-EAS293:2:2:590:480#0/1    ---------------------------AGTTCAACTGATTGAAC------------------------------------------------------------
  @HWI-EAS293:2:2:1398:1075#0/1    ---------------------------AGTTCAACTGATTGAAC------------------------------------------------------------
  @HWI-EAS293:2:2:1539:1926#0/1    ---------------------------AGTTCAACTGATTGAAC------------------------------------------------------------
   @HWI-EAS293:2:3:858:1337#0/1    ---------------------------AGTTCAACTGATTGAAC------------------------------------------------------------
   @HWI-EAS293:2:4:342:1680#0/1    ---------------------------AGcTCAACTGATTGAAC------------------------------------------------------------
   @HWI-EAS293:2:4:957:1305#0/1    ---------------------------AGTTCAACTGATTGAAC------------------------------------------------------------
    @HWI-EAS293:2:4:993:955#0/1    ---------------------------AGTTCAACTGATTGAAC------------------------------------------------------------
   @HWI-EAS293:2:4:1632:985#0/1    ---------------------------AGTTCAACTGATTGAAC------------------------------------------------------------
   @HWI-EAS293:2:4:1769:322#0/1    ---------------------------AGcTCAACTGATTGAAC------------------------------------------------------------
    @HWI-EAS293:2:5:220:644#0/1    ---------------------------AGTTCAACTGATTGAAC------------------------------------------------------------
    @HWI-EAS293:2:5:380:818#0/1    ---------------------------AGTTCAACTGATTGAAC------------------------------------------------------------
   @HWI-EAS293:2:5:386:1500#0/1    ---------------------------AGTTCAACTGATTGAAC------------------------------------------------------------
   @HWI-EAS293:2:5:774:1023#0/1    ---------------------------AGTTCAACTGATTGAAC------------------------------------------------------------
   @HWI-EAS293:2:5:1002:568#0/1    ---------------------------AGTTCAACTGATTGAAC------------------------------------------------------------
   @HWI-EAS293:2:5:1482:821#0/1    ---------------------------AGTTCAACTGATTGAAC------------------------------------------------------------
  @HWI-EAS293:2:5:1660:1470#0/1    ---------------------------AGTTCAACTGATTGAAC------------------------------------------------------------
    @HWI-EAS293:2:6:289:302#0/1    ---------------------------AGcTCAACTGATTGAAC------------------------------------------------------------
  @HWI-EAS293:2:6:1765:1069#0/1    ---------------------------AGTTCAACTGATTGAAC------------------------------------------------------------
    @HWI-EAS293:2:7:262:252#0/1    ---------------------------AGTTCAACTGATTGAAt------------------------------------------------------------
     @HWI-EAS293:2:7:537:73#0/1    ---------------------------AGcTCAACTGATTGAAC------------------------------------------------------------
    @HWI-EAS293:2:7:582:739#0/1    ---------------------------AGcTCAACTGATTGAAC------------------------------------------------------------
    @HWI-EAS293:2:7:721:183#0/1    ---------------------------AGTTCAACTGATTGAAC------------------------------------------------------------
    @HWI-EAS293:2:8:55:1687#0/1    ---------------------------AGTTCAACTGATTGAAC------------------------------------------------------------
   @HWI-EAS293:2:8:871:1945#0/1    ---------------------------AGTTCAACTGATTGAAC------------------------------------------------------------
  @HWI-EAS293:2:8:1377:1389#0/1    ---------------------------AGTTCAACTGATTGAAC------------------------------------------------------------
   @HWI-EAS293:2:8:1662:707#0/1    ---------------------------AGTTCAACTGATTGAAC------------------------------------------------------------
  @HWI-EAS293:2:8:1728:1985#0/1    ---------------------------AGTTCAACTGATTGAAC------------------------------------------------------------
    @HWI-EAS293:2:8:1750:21#0/1    ---------------------------AGTTCAACTGATTGAAC------------------------------------------------------------
   @HWI-EAS293:2:9:771:1136#0/1    ---------------------------AGTTCAACTGATTGAAC------------------------------------------------------------
   @HWI-EAS293:2:9:1502:870#0/1    ---------------------------AGcTCAACTGATTGAAC------------------------------------------------------------
   @HWI-EAS293:2:9:1564:700#0/1    ---------------------------AGTTCAACTGATTGAAC------------------------------------------------------------
  @HWI-EAS293:2:10:253:1059#0/1    ---------------------------AGcTCAACTGATTGAAC------------------------------------------------------------
   @HWI-EAS293:2:10:709:520#0/1    ---------------------------AGTTCAACTGATTGAAC------------------------------------------------------------
 @HWI-EAS293:2:10:1440:1590#0/1    ---------------------------AGTTCAACTGATTGAAC------------------------------------------------------------
 @HWI-EAS293:2:10:1720:1174#0/1    ---------------------------AGcTCAACTGATTGAAC------------------------------------------------------------
  @HWI-EAS293:2:11:180:2004#0/1    ---------------------------AGcTCAACTGATTGAAC------------------------------------------------------------
   @HWI-EAS293:2:11:816:456#0/1    ---------------------------AGcTCAACTGATTGAAC------------------------------------------------------------
  @HWI-EAS293:2:11:1039:373#0/1    ---------------------------AGTTCAACTGATTGAAC------------------------------------------------------------
  @HWI-EAS293:2:11:1135:511#0/1    ---------------------------AGcTCAACTGATTGAAC------------------------------------------------------------
  @HWI-EAS293:2:11:1262:458#0/1    ---------------------------AGTTCAACTGATTGAAC------------------------------------------------------------
    @HWI-EAS293:2:12:55:636#0/1    ---------------------------AGTTCAACTGATTGAAC------------------------------------------------------------
   @HWI-EAS293:2:12:967:166#0/1    ---------------------------AGcTCAACTGATTGAAC------------------------------------------------------------
 @HWI-EAS293:2:12:1169:1652#0/1    ---------------------------AGcTCAACTGATTGAAC------------------------------------------------------------
  @HWI-EAS293:2:12:1642:797#0/1    ---------------------------AGTTCAACTGATTGAAC------------------------------------------------------------
   @HWI-EAS293:2:13:65:1081#0/1    ---------------------------AGTTCAACTGATTGAAC------------------------------------------------------------
  @HWI-EAS293:2:13:187:1347#0/1    ---------------------------AGcTCAACTGATTGAAC------------------------------------------------------------
    @HWI-EAS293:2:13:331:29#0/1    ---------------------------AGTTCAACTGATTGAAC------------------------------------------------------------
   @HWI-EAS293:2:13:536:662#0/1    ---------------------------AGcTCAACTGATTGAAC------------------------------------------------------------
  @HWI-EAS293:2:13:1037:697#0/1    ---------------------------AGTTCAACTGATTGAAC------------------------------------------------------------
  @HWI-EAS293:2:13:1380:206#0/1    ---------------------------AGTTCAACTGATTGAAC------------------------------------------------------------
   @HWI-EAS293:2:14:946:983#0/1    ---------------------------AGTTCAACTGATTGAAC------------------------------------------------------------
    @HWI-EAS293:2:1:746:362#0/1    -------------------------------------------------------------------------ATCGAAATTAGTCTTTCGCTCCTATCTGAAC
   @HWI-EAS293:2:1:1222:530#0/1    -------------------------------------------------------------------------ATCGAAATTAGTCTTTCGCTCCTATCTGAAC
   @HWI-EAS293:2:2:952:1829#0/1    -------------------------------------------------------------------------ATCGAAATTAGcCTTTCGCTCCTATCTGAAC
  @HWI-EAS293:2:3:1380:1053#0/1    -------------------------------------------------------------------------ATCGAAATTAGTCTTTCGCTCCTATCTGAAC
  @HWI-EAS293:2:3:1542:1934#0/1    -------------------------------------------------------------------------ATCGAAATTAGTCTTTCGCTCCTATCTGAAC
    @HWI-EAS293:2:6:509:569#0/1    -------------------------------------------------------------------------ATCGAAATTAGTCTTTCGCTCCTATCTGAAC
   @HWI-EAS293:2:6:734:1526#0/1    -------------------------------------------------------------------------ATCGAAATTAGTCTTTCGCTCCTATCTGAAC
   @HWI-EAS293:2:6:903:1982#0/1    -------------------------------------------------------------------------ATCGAAATTAGTCTTTCGCTCCTATCTGAAC
   @HWI-EAS293:2:6:1658:945#0/1    -------------------------------------------------------------------------ATCGAAATTAGTCTTTCGCTCCTATCTGAAC
    @HWI-EAS293:2:7:125:155#0/1    -------------------------------------------------------------------------ATCGAAATTAGTCTTTCGCTCCTATCTGAAC
    @HWI-EAS293:2:7:758:661#0/1    -------------------------------------------------------------------------ATCGAAATTAGTCTTTCGCTCCTATCTGAAC
   @HWI-EAS293:2:7:967:1109#0/1    -------------------------------------------------------------------------ATCGAAATTAGTCTTTCGCTCCTATCTGAAC
   @HWI-EAS293:2:8:123:1819#0/1    -------------------------------------------------------------------------ATCGAAATTAGTCTTTCGCTCCTATCTGAAC
   @HWI-EAS293:2:8:1435:705#0/1    -------------------------------------------------------------------------ATCGAAATTAGTCTTTCGCTCCTATCTGAAC
  @HWI-EAS293:2:9:1496:1737#0/1    -------------------------------------------------------------------------ATCGAAATTAGTCTTTCGCTCCTATCTGAAC
   @HWI-EAS293:2:10:236:759#0/1    -------------------------------------------------------------------------ATCGAAATTAGTCTTTCGCTCCTATCTGAAC
  @HWI-EAS293:2:10:871:1032#0/1    -------------------------------------------------------------------------ATCGAAATTAGTCTTTCGCTCCTATCTGAAC
 @HWI-EAS293:2:11:1386:1840#0/1    -------------------------------------------------------------------------ATCGAAATTAGTCTTTCGCTCCTATCTGAAC
  @HWI-EAS293:2:12:1086:606#0/1    -------------------------------------------------------------------------ATCGAAATTAGTCTTTCGCTCCTATCTGAAC
  @HWI-EAS293:2:14:1214:367#0/1    -------------------------------------------------------------------------ATCGAAATTAGTCTTTCGCTCCTATCTGAAC
   @HWI-EAS293:2:1:154:1805#0/1    ---------------------------------------------------------------------------CGAAATTAGTCTTTCGCTCCTATCTGAAC
   @HWI-EAS293:2:1:801:1619#0/1    ---------------------------------------------------------------------------CGAAATTAGTCTTTCGCTCCTATCTGAAC
   @HWI-EAS293:2:1:1242:277#0/1    ---------------------------------------------------------------------------CGAAATTAGTCTTTCGCTCCTATCTGAAC
    @HWI-EAS293:2:7:715:667#0/1    ---------------------------------------------------------------------------CGAAATTAGTCTTTCGCTCCTATCTGAAC
  @HWI-EAS293:2:8:1761:1380#0/1    ---------------------------------------------------------------------------CGAAATTAGTCTTTCGCTCCTATCTGAAC
    @HWI-EAS293:2:9:492:477#0/1    ---------------------------------------------------------------------------CGAAATTAGTCTTTCGCTCCTATCTGAAC
  @HWI-EAS293:2:9:1638:1164#0/1    ---------------------------------------------------------------------------CGAAATTAGTCTTTCGCTCCTATCTGAAC
  @HWI-EAS293:2:10:227:1009#0/1    ---------------------------------------------------------------------------CGAAATTAGTCTTTCGCTCCTATCTGAAC
   @HWI-EAS293:2:11:53:1303#0/1    ---------------------------------------------------------------------------CGAAATTAGTCTTTCGCTCCTATCTGAAC
   @HWI-EAS293:2:11:1489:56#0/1    ---------------------------------------------------------------------------CGAAATTAGTCTTTCGCTCCTATCTGAAC
 @HWI-EAS293:2:11:1577:1110#0/1    ---------------------------------------------------------------------------CGAAATTAGTCTTTCGCTCCTATCTGAAC
 @HWI-EAS293:2:13:1592:1901#0/1    ---------------------------------------------------------------------------CGAAATTAGTCTTTCGCTCCTATCTGAAC
   @HWI-EAS293:2:14:18:1083#0/1    ---------------------------------------------------------------------------CGAAATTAGTCTTTCGCTCCTATCTGAAC
  @HWI-EAS293:2:14:497:1987#0/1    ---------------------------------------------------------------------------CGAAATTAGTCTTTCGCTCCTATCTGAAC
```

```
@HWI-EAS293:2:4:271:836#0/1      ----------------------------------------------------------------------------GAAATTAGTCTTTCGCTCCTATCTGAAC
@HWI-EAS293:2:4:1298:1981#0/1    ----------------------------------------------------------------------------GAAATTAGTCTTTCGCTCCTATCTGAAC
@HWI-EAS293:2:5:1602:668#0/1     ----------------------------------------------------------------------------GAAATTAGTCTTTCGCTCCTATCTGAAC
@HWI-EAS293:2:6:125:203#0/1      ----------------------------------------------------------------------------GAAATTAGTCTTTCGCTCCTATCTGAAC
@HWI-EAS293:2:9:763:1668#0/1     ----------------------------------------------------------------------------GAAATTAGTCTTTCGCTCCTATCTGAAC
@HWI-EAS293:2:10:1238:1967#0/1   ----------------------------------------------------------------------------GAAATTAGTCTTTCGCTCCTATCTGAAC
@HWI-EAS293:2:2:1134:15#0/1      -----------------------------------------------------------------------------gAATTgGTCTTTCGCTCCTATCTGAAC
@HWI-EAS293:2:12:1170:546#0/1    -----------------------------------------------------------------------------gAATTAGTCTTTCGCTCCTATCTGAAC
@HWI-EAS293:2:1:284:736#0/1      ----------------------------------------------------------------------------------AGTCTTTCGCTCCTATCTGAAC
@HWI-EAS293:2:1:322:667#0/1      ----------------------------------------------------------------------------------AGTCTTTCGCTCCTATCTGAAC
@HWI-EAS293:2:1:918:505#0/1      ----------------------------------------------------------------------------------AGTtTTTCGCTCCTATCTGAAC
@HWI-EAS293:2:1:1705:276#0/1     ----------------------------------------------------------------------------------AGTCTTTCGCTCCTATCTGAAC
@HWI-EAS293:2:2:83:1918#0/1      ----------------------------------------------------------------------------------AGTtTTTCGCTCCTATCTGAAC
@HWI-EAS293:2:2:371:589#0/1      ----------------------------------------------------------------------------------AGTCTTTCGCTCCTATCTGAAC
@HWI-EAS293:2:2:1275:777#0/1     ----------------------------------------------------------------------------------AGTtTTTCGCTCCTATCaGAAC
@HWI-EAS293:2:2:1399:1956#0/1    ----------------------------------------------------------------------------------AGTtTTTCGCTCCTATCTGAAC
@HWI-EAS293:2:3:118:1073#0/1     ----------------------------------------------------------------------------------AGTtTTTCGCTCCTATCTGAAC
@HWI-EAS293:2:3:128:180#0/1      ----------------------------------------------------------------------------------AGTtTTTCGCTCCTATCTGAAC
@HWI-EAS293:2:3:469:1522#0/1     ----------------------------------------------------------------------------------AGTCTTTCGCTCCTATCTGAAC
@HWI-EAS293:2:3:729:1839#0/1     ----------------------------------------------------------------------------------AGTCTTTCGCTCCTATCTGAAC
@HWI-EAS293:2:3:750:810#0/1      ----------------------------------------------------------------------------------AGTCTTTCGCTCCTATCTGAAC
@HWI-EAS293:2:3:1059:933#0/1     ----------------------------------------------------------------------------------AGTtTTTCGCTCCTATCTGAAC
@HWI-EAS293:2:3:1277:84#0/1      ----------------------------------------------------------------------------------AGTCTTTCGCTCCTATCTGAAC
@HWI-EAS293:2:4:163:1100#0/1     ----------------------------------------------------------------------------------AGTtTTTCGCTCCTATCTGAAC
@HWI-EAS293:2:4:1215:560#0/1     ----------------------------------------------------------------------------------AGTtTTTCGCTCCTATCTGAAC
@HWI-EAS293:2:5:710:1639#0/1     ----------------------------------------------------------------------------------AGTtTTTCGCTCCTATCTGAAC
@HWI-EAS293:2:5:1076:1030#0/1    ----------------------------------------------------------------------------------AGTtTTTCGCTCCTATCTGAAC
@HWI-EAS293:2:5:1221:209#0/1     ----------------------------------------------------------------------------------AGTCTTTCGCTCCTATCTGAAC
@HWI-EAS293:2:5:1302:1180#0/1    ----------------------------------------------------------------------------------AGTCTTTCGCTCCTATCTGAAC
@HWI-EAS293:2:6:14:128#0/1       ----------------------------------------------------------------------------------AGTtTTTCGCTCCTATCTGAAC
@HWI-EAS293:2:6:615:876#0/1      ----------------------------------------------------------------------------------AGTtTTTCGCTCCTATCTGAAC
@HWI-EAS293:2:6:705:1326#0/1     ----------------------------------------------------------------------------------AGTCTTTCGCTCCTATCTGAAC
@HWI-EAS293:2:6:1368:78#0/1      ----------------------------------------------------------------------------------AGTCTTTCGCTCCTATCTGAAC
@HWI-EAS293:2:6:1662:1528#0/1    ----------------------------------------------------------------------------------AGTCTTTCGCTCCTATCTGAAC
@HWI-EAS293:2:7:1238:964#0/1     ----------------------------------------------------------------------------------AGTCTTTCGCTCCTATCTGAAC
@HWI-EAS293:2:7:1271:285#0/1     ----------------------------------------------------------------------------------AGTtTTTCGCTCCTATCTGAAC
@HWI-EAS293:2:7:1570:315#0/1     ----------------------------------------------------------------------------------AGTCTTTCGCTCCTATCTGAAC
@HWI-EAS293:2:8:249:1286#0/1     ----------------------------------------------------------------------------------AGTCTTTCGCTCCTATCTGAAC
@HWI-EAS293:2:8:391:1667#0/1     ----------------------------------------------------------------------------------AGTCTTTCGCTCCTATCTGAAC
@HWI-EAS293:2:8:576:665#0/1      ----------------------------------------------------------------------------------AGTCTTTCGCTCCTATCTGAAC
@HWI-EAS293:2:8:1120:25#0/1      ----------------------------------------------------------------------------------AGTtTTTCGCTCCTATCTGAAC
@HWI-EAS293:2:9:631:1022#0/1     ----------------------------------------------------------------------------------AGTtTTTCGCTCCTATCTGAAC
@HWI-EAS293:2:9:887:2035#0/1     ----------------------------------------------------------------------------------AGTtTTTCGCTCCTATCTGAAC
@HWI-EAS293:2:9:1087:938#0/1     ----------------------------------------------------------------------------------AGTtTTTCGCTCCTATCTGAAC
@HWI-EAS293:2:9:1237:838#0/1     ----------------------------------------------------------------------------------AGTCTTTCGCTCCTATCTGAAC
@HWI-EAS293:2:10:157:1747#0/1    ----------------------------------------------------------------------------------AGTtTTTCGCTCCTATCTGAAC
@HWI-EAS293:2:10:659:1671#0/1    ----------------------------------------------------------------------------------AGTtTTTCGCTCCTATCTGAAC
@HWI-EAS293:2:10:1528:930#0/1    ----------------------------------------------------------------------------------AGTCTTTCGCTCCTATCTGAAC
@HWI-EAS293:2:10:1693:422#0/1    ----------------------------------------------------------------------------------AGTCTTTCGCTCCTATCTGAAC
@HWI-EAS293:2:11:668:497#0/1     ----------------------------------------------------------------------------------AGTtTTTCGCTCCTATCTGAAC
@HWI-EAS293:2:11:1074:545#0/1    ----------------------------------------------------------------------------------AGTtTTTCGCTCCTATCTGAAC
@HWI-EAS293:2:11:1707:613#0/1    ----------------------------------------------------------------------------------AGTCTTTCGCTCCTATCTGAAC
@HWI-EAS293:2:12:744:1178#0/1    ----------------------------------------------------------------------------------AGTtTTTCGCTCCTATCTGAAC
@HWI-EAS293:2:12:763:1114#0/1    ----------------------------------------------------------------------------------AGTtTTTCGCTCCTATCTGAAC
@HWI-EAS293:2:12:1489:380#0/1    ----------------------------------------------------------------------------------AGTtTTTCGCTCCTATCTGAAC
@HWI-EAS293:2:12:1679:156#0/1    ----------------------------------------------------------------------------------AGTtTTTCGCTCCTATCTGAAC
@HWI-EAS293:2:12:1786:1532#0/1   ----------------------------------------------------------------------------------AGTtTTTCGCTCCTATCTGAAC
@HWI-EAS293:2:13:16:1704#0/1     ----------------------------------------------------------------------------------AGTCTTTCGCTCCTATCTGAAC
@HWI-EAS293:2:13:435:1354#0/1    ----------------------------------------------------------------------------------AGTtTTTCGCTCCTATCTGAAC
@HWI-EAS293:2:13:964:1718#0/1    ----------------------------------------------------------------------------------AGTtTTTCGCTCCTATCTGAAC
@HWI-EAS293:2:13:1561:1639#0/1   ----------------------------------------------------------------------------------AGTtTTTCGCTCCTATCTGAAC
@HWI-EAS293:2:14:73:524#0/1      ----------------------------------------------------------------------------------AGTtTTTCGCTCCTATCTGAAC
@HWI-EAS293:2:14:103:531#0/1     ----------------------------------------------------------------------------------AGTCTTTCGCTCCTATCTGAAC
@HWI-EAS293:2:14:728:357#0/1     ----------------------------------------------------------------------------------AGTtTTTCGCTCCTATCTGAAC
@HWI-EAS293:2:14:821:1176#0/1    ----------------------------------------------------------------------------------AGTCTTTCGCTCCTATCTGAAC
@HWI-EAS293:2:14:1094:1668#0/1   ----------------------------------------------------------------------------------AGTtTTTCGCTCCTATCTGAAC
@HWI-EAS293:2:1:708:1420#0/1     -------------------------------------------------------------------------------------CTTTCGCTCCTATCTGAAC
@HWI-EAS293:2:1:982:351#0/1      -------------------------------------------------------------------------------------CTTTCGCTCCTATCTGAAC
@HWI-EAS293:2:8:1098:737#0/1     -------------------------------------------------------------------------------------CTTTCGCTCCTATCTGAAC
@HWI-EAS293:2:10:1024:1576#0/1   -------------------------------------------------------------------------------------CTTTCGCTCCTATCTGAAC
@HWI-EAS293:2:10:1629:1588#0/1   -------------------------------------------------------------------------------------CTTTCGCTCCTATCTGAAC
@HWI-EAS293:2:12:1699:218#0/1    -------------------------------------------------------------------------------------CTTTCGCTCCTATCTGAAC
```

snR72

```
                       1    .........|.........|.........|.........|.........|.........|.........|.........|.........|........ 98
                   snR72    GTGATGTGATGACAACTTCTTGAGCTATATATTTTCTTGAGAACATCAATGAAGAAAACGTCTCATCAAATGATTTGCACGTCAGTCTGATCACAAGC
@HWI-EAS293:2:4:1475:1993#0/1    ---------------------GAGCTATATATTTTCTTGAGAAC------------------------------------------------------
@HWI-EAS293:2:7:210:1603#0/1    ---------------------GAGCTATATATTTTCTTGAGAAC------------------------------------------------------
@HWI-EAS293:2:9:939:1164#0/1    ---------------------GAGCTATATATTTTCTTGAGAAC------------------------------------------------------
@HWI-EAS293:2:13:409:1113#0/1    ---------------------GAGCTATATATTTTCTTGAGAAC------------------------------------------------------
@HWI-EAS293:2:1:1349:1929#0/1    -------------------------TATATATTTTCTTGAGAAC------------------------------------------------------
@HWI-EAS293:2:2:1069:932#0/1    -------------------------TATATATTTTCTTGAGAAC------------------------------------------------------
@HWI-EAS293:2:3:1333:901#0/1    -------------------------TATATATTTTCTTGAGAAC------------------------------------------------------
@HWI-EAS293:2:5:175:1922#0/1    -------------------------TATATATTTTCTTGAGAAC------------------------------------------------------
@HWI-EAS293:2:6:224:1158#0/1    -------------------------TATATATTTTCTTGAGAAC------------------------------------------------------
@HWI-EAS293:2:6:449:1925#0/1    -------------------------TATATATTTTCTTGAGAAC------------------------------------------------------
@HWI-EAS293:2:9:1365:312#0/1    -------------------------TATATATTTTCTTGAGAAC------------------------------------------------------
@HWI-EAS293:2:10:408:1055#0/1    -------------------------TATATATTTTCTTGAGAAC------------------------------------------------------
@HWI-EAS293:2:12:395:266#0/1    -------------------------TATATATTTTCTTGAGAAC------------------------------------------------------
@HWI-EAS293:2:12:470:2013#0/1    -------------------------TATATATTTTCTTGAGAAC------------------------------------------------------
@HWI-EAS293:2:13:635:563#0/1    -------------------------TATATATTTTCTTGAGAAC------------------------------------------------------
@HWI-EAS293:2:16:677:652#0/1    -------------------------TATATATTTTCTTGAGAAC------------------------------------------------------
@HWI-EAS293:2:16:1224:123#0/1    -------------------------TATATATTTTCTTGAGAAC------------------------------------------------------
@HWI-EAS293:2:19:105:1992#0/1    -------------------------TATATATTTTCTTGAGAAC------------------------------------------------------
@HWI-EAS293:2:8:1080:1405#0/1    --------------------------------------------ATCAATGAAGAAAACGTCTCATCAAATGATTTGCACGTCAGTCTGATCACA---
@HWI-EAS293:2:10:83:1966#0/1    --------------------------------------------ATCAATGAAGAAAACGTCTCATCAAATGATTTGCACGTCAGTCTGATCACA---
@HWI-EAS293:2:4:36:1329#0/1    -----------------------------------------------------------GTCTCATCAAATGATTTGCACGTCAGTCTGATCACA---
@HWI-EAS293:2:4:374:389#0/1    -----------------------------------------------------------GTCTCATCAAATGATTTGCACGTCAGTCTGATCACA---
@HWI-EAS293:2:6:315:610#0/1    -----------------------------------------------------------GTCTCATCAAATGATTTGCACGTCAGTCTGATCACA---
@HWI-EAS293:2:7:141:1260#0/1    -----------------------------------------------------------GTCTCATCAAATGATTTGCACGTCAGTCTGATCACA---
@HWI-EAS293:2:8:863:1877#0/1    -----------------------------------------------------------GTCTCATCAAATGATTTGCACGTCAGTCTGATCACA---
@HWI-EAS293:2:9:25:1329#0/1    -----------------------------------------------------------GTCTCATCAAATGATTTGCACGTCAGTCTGATCAC----
@HWI-EAS293:2:9:1322:1523#0/1    -----------------------------------------------------------GTCTCATCAAATGATTTGCAaGTCAGTCTGATCACA---
@HWI-EAS293:2:12:105:1267#0/1    -----------------------------------------------------------GTCTCATCAAATGATTTGCACGTCAGTCTGATCACA---
@HWI-EAS293:2:17:1604:513#0/1    -----------------------------------------------------------GTCTCATCAAATGATTTGCACGTCAGTCTGATCACA---
@HWI-EAS293:2:18:110:809#0/1    -----------------------------------------------------------GTCTCATCAAATGATTTGCACGTCAGTCTGATCACA---
@HWI-EAS293:2:1:246:1693#0/1    ----------------------------------------------------------------ATCAAATGATTTGCACGTCAGTCTGATCAC----
@HWI-EAS293:2:1:1466:1752#0/1    ----------------------------------------------------------------ATCAAATGATTTGCACGTCAGTCTGATCACA---
@HWI-EAS293:2:1:1592:510#0/1    ----------------------------------------------------------------ATCAAATGATTTGCACGTCAGTCTGATC------
@HWI-EAS293:2:1:1646:1326#0/1    ----------------------------------------------------------------ATCAAATGATTTGCACGTCAGTCTGATCACA---
@HWI-EAS293:2:1:1665:933#0/1    ----------------------------------------------------------------ATCAAATGATTTGCACGTCAGTCTGATCACA---
@HWI-EAS293:2:1:1770:1007#0/1    ----------------------------------------------------------------ATCAAATGATTTGCACGTCAGTCTGATCACA---
@HWI-EAS293:2:2:147:1474#0/1    ----------------------------------------------------------------ATCAAATGATTTGCACGTCAGTCTGATCAC----
@HWI-EAS293:2:2:1201:562#0/1    ----------------------------------------------------------------ATCAAATGATTTGCACGTCAGTCTGATCAC----
@HWI-EAS293:2:2:1663:298#0/1    ----------------------------------------------------------------ATCAAATGATTTGCACGTCAGTCTGATCAC----
@HWI-EAS293:2:3:929:1185#0/1    ----------------------------------------------------------------ATCAAATGATTTGCACGTCAGTCTGATCACA---
@HWI-EAS293:2:3:941:1390#0/1    ----------------------------------------------------------------ATCAAATGATTTGCACGcCAGTCTGATCACA---
@HWI-EAS293:2:3:1173:1442#0/1    ----------------------------------------------------------------ATCAAATGATTTGCACGTCAGTCTGATCAC----
@HWI-EAS293:2:3:1564:614#0/1    ----------------------------------------------------------------ATCAAATGATTTGCACGTCAGTCTGATCACA---
@HWI-EAS293:2:4:411:821#0/1    ----------------------------------------------------------------ATCAAATGATTTGCACGTCAGTCTGATCACA---
@HWI-EAS293:2:4:459:1920#0/1    ----------------------------------------------------------------ATCAAATGATTTGCACGTCAGTCTGATCACA---
@HWI-EAS293:2:4:502:711#0/1    ----------------------------------------------------------------ATCAAATGATTTGCACGTCAGTCTGATCAaA---
@HWI-EAS293:2:4:1164:461#0/1    ----------------------------------------------------------------ATCAAATGATTTGCACGTCAGTCTGATC------
@HWI-EAS293:2:5:106:33#0/1    ----------------------------------------------------------------ATCAAATGATTTGCACGTCAGTCTGATCACA---
@HWI-EAS293:2:5:293:1583#0/1    ----------------------------------------------------------------ATCAAATGATTTGCACGTCAGTCTGATC------
@HWI-EAS293:2:5:583:117#0/1    ----------------------------------------------------------------ATCAAATGATTTGCACGTCAGTCTGATC------
@HWI-EAS293:2:5:735:1971#0/1    ----------------------------------------------------------------ATCAAATGATTTGCACGTCAGTCTGATCACA---
@HWI-EAS293:2:5:854:1838#0/1    ----------------------------------------------------------------ATCAAATGATTTGCACGTCAGTCTGATC------
@HWI-EAS293:2:5:1018:44#0/1    ----------------------------------------------------------------ATCAAATGATTTGCACGTCAGTCTGATC------
@HWI-EAS293:2:6:479:484#0/1    ----------------------------------------------------------------ATCAAATGATTTGCACGTCAGTCTGATC------
@HWI-EAS293:2:6:1538:1914#0/1    ----------------------------------------------------------------ATCAAATGATTTGCACGTCAGTCTGATCAC----
@HWI-EAS293:2:7:261:1961#0/1    ----------------------------------------------------------------ATCAAATGATTTGCACGTCAGTCTGATCACA---
@HWI-EAS293:2:7:870:1953#0/1    ----------------------------------------------------------------ATCAAATGATTTGCACGTCAGTCTGATC------
@HWI-EAS293:2:7:881:1534#0/1    ----------------------------------------------------------------ATCAAATGATTTGCACGTCAGTCTGATC------
@HWI-EAS293:2:7:1218:1345#0/1    ----------------------------------------------------------------ATCAAATGATTTGCACGTCAGTCTGATCACA---
@HWI-EAS293:2:7:1272:95#0/1    ----------------------------------------------------------------ATCAAATGATTTGCACGTCAGTCTGATCAC----
@HWI-EAS293:2:7:1338:725#0/1    ----------------------------------------------------------------ATCAAATGATTTGCACGTCAGTCTGATCACA---
@HWI-EAS293:2:7:1400:236#0/1    ----------------------------------------------------------------ATCAAATGATTTGCACGTCAGTCTGATC------
@HWI-EAS293:2:7:1535:629#0/1    ----------------------------------------------------------------ATCAAATGATTTGCACGTCAGTCTGATCACA---
@HWI-EAS293:2:8:350:155#0/1    ----------------------------------------------------------------ATCAAATGATTTGCACGTCAGTCTGATCACA---
@HWI-EAS293:2:8:812:684#0/1    ----------------------------------------------------------------ATCAAATGATTTGCACGTCAGTCTGATCACA---
@HWI-EAS293:2:8:1048:1512#0/1    ----------------------------------------------------------------ATCAAATGATTTGCACGTCAGTCTGATCACA---
@HWI-EAS293:2:8:1456:526#0/1    ----------------------------------------------------------------ATCAAATGATTTGCACGTCAGTCTGATC------
@HWI-EAS293:2:9:1179:1104#0/1    ----------------------------------------------------------------ATCAAATGATTTGCACGTCAGTCTGATCACA---
@HWI-EAS293:2:9:1276:1876#0/1    ----------------------------------------------------------------ATCAAATGATTTGCACGTCAGTCTGcTCACA---
@HWI-EAS293:2:9:1409:100#0/1    ----------------------------------------------------------------ATCAAATGATTTGCACGTCAGTCTGATCACA---
@HWI-EAS293:2:9:1604:1672#0/1    ----------------------------------------------------------------ATCAAATGATTTGCACGTCAGTCTGATCACA---
@HWI-EAS293:2:10:177:552#0/1    ----------------------------------------------------------------ATCAAATGATTTGCACGTCAGTCTGATCACA---
@HWI-EAS293:2:10:702:1702#0/1    ----------------------------------------------------------------ATCAAATGATTTGCACGTCAGTCTGATCACA---
@HWI-EAS293:2:10:1721:826#0/1    ----------------------------------------------------------------ATCAAATGATTTGCACGTCAGTCTGATC------
@HWI-EAS293:2:12:414:1211#0/1    ----------------------------------------------------------------ATCAAATGATTTGCACGTCAGTCTGATCAC----
@HWI-EAS293:2:12:625:1427#0/1    ----------------------------------------------------------------ATCAAATGATTTGCACGTCAGTCTGATCACA---
@HWI-EAS293:2:12:916:1788#0/1    ----------------------------------------------------------------ATCAAATGATTTGCACGTCAGTCTGATCcCA---
@HWI-EAS293:2:12:1082:1165#0/1    ----------------------------------------------------------------ATCAAATGATTTGCACGTCAGTCTGATCACA---
@HWI-EAS293:2:12:1414:1138#0/1    ----------------------------------------------------------------ATCAAATGATTTGCACGTCAGTCTGATCACA---
@HWI-EAS293:2:13:902:781#0/1    ----------------------------------------------------------------ATCAAATGATTTGCACGTCAGTCTGATCAC----
@HWI-EAS293:2:13:1031:474#0/1    ----------------------------------------------------------------ATCAAATGATTTGCACGTCAGTCTGATCAC----
@HWI-EAS293:2:13:1556:978#0/1    ----------------------------------------------------------------ATCAAATGATTTGCACGTCAGTCTGATCACA---
@HWI-EAS293:2:14:317:1916#0/1    ----------------------------------------------------------------ATCAAATGATTTGCACGTCAGTCTGATC------
@HWI-EAS293:2:14:627:1222#0/1    ----------------------------------------------------------------ATCAAA-GATTTGCACGTCAGTCTGATCACA---
@HWI-EAS293:2:14:1010:307#0/1    ----------------------------------------------------------------ATCAAATGATTTGCACGTCAGTCTGATC------
@HWI-EAS293:2:15:1131:1117#0/1    ----------------------------------------------------------------ATCAAATGATTTGCACGTCAGTCTGATC------
@HWI-EAS293:2:15:1213:433#0/1    ----------------------------------------------------------------ATCAAATGATTTGCACGTCAGTCTGATCACA---
@HWI-EAS293:2:16:251:1748#0/1    ----------------------------------------------------------------ATCAAATGATTTGCACGTCAGTCTGATCACA---
@HWI-EAS293:2:16:589:1162#0/1    ----------------------------------------------------------------ATCAAATGATTTGCACGTCAGTCTGATCACA---
@HWI-EAS293:2:16:628:626#0/1    ----------------------------------------------------------------ATCAAATGATTTGCACGTCAGTCTGATCACA---
@HWI-EAS293:2:16:956:1551#0/1    ----------------------------------------------------------------ATCAAATGATTTGCACGTCAGTCTGATC------
@HWI-EAS293:2:16:1242:635#0/1    ----------------------------------------------------------------ATCAAATGATTTGCACGTCAGTCTGATCAC----
@HWI-EAS293:2:16:1458:552#0/1    ----------------------------------------------------------------ATCAAATGATTTGCACGTCAGTCTGATC------
@HWI-EAS293:2:16:1690:1731#0/1    ----------------------------------------------------------------ATCAAATGATTTGCACGTCAGTCTGATC------
@HWI-EAS293:2:17:201:167#0/1    ----------------------------------------------------------------ATCAAATGATTTGCACGTCAGTCTGATCACA---
@HWI-EAS293:2:17:958:1841#0/1    ----------------------------------------------------------------ATCAAATGATTTGCACGTCAGTCTGATCAC----
@HWI-EAS293:2:17:1196:847#0/1    ----------------------------------------------------------------ATCAAATGATTTGCACGTCAGTCTGATCACA---
@HWI-EAS293:2:18:167:99#0/1    ----------------------------------------------------------------ATCAAATGATTTGCACGTCAGTCTGATCAC----
@HWI-EAS293:2:18:170:1725#0/1    ----------------------------------------------------------------ATCAAATGATTTGCACGTCAGTCTGATCACA---
@HWI-EAS293:2:18:512:1686#0/1    ----------------------------------------------------------------ATCAAATGATTTGCACGTCAGTCTGATCACA---
@HWI-EAS293:2:18:710:418#0/1    ----------------------------------------------------------------ATCAAATGATTTGCACGTCAGTCTGATCAC----
@HWI-EAS293:2:18:1035:932#0/1    ----------------------------------------------------------------gTCAAATGATTTGCACGTCAGTCTGATCACA---
@HWI-EAS293:2:18:1190:1617#0/1    ----------------------------------------------------------------ATCAAATGATTTGCACGTCAGTCTGATCACA---
@HWI-EAS293:2:18:1653:363#0/1    ----------------------------------------------------------------ATCAAATGATTTGCACGTCAGTCTGATCACA---
@HWI-EAS293:2:19:181:1846#0/1    ----------------------------------------------------------------ATCAAATGATTTGCACGTCAGTCTGATCACA---
@HWI-EAS293:2:1:1168:143#0/1    -----------------------------------------------------------------TCAAATGATTTGCACGTCAGTCTGATCACA---
@HWI-EAS293:2:1:1239:1306#0/1    -----------------------------------------------------------------TCAAATGATTTGCACGTCAGTCTGATCACA---
@HWI-EAS293:2:3:1404:1885#0/1    -----------------------------------------------------------------TCAAATGATTTGCACGTCAGTCTGATCACA---
@HWI-EAS293:2:4:1582:1256#0/1    -----------------------------------------------------------------TCAAATGATTTGCACGTCAGTCTGATCACA---
@HWI-EAS293:2:4:1604:1582#0/1    -----------------------------------------------------------------TCAAATGATTTGCACGTCAGTCTGATCACA---
@HWI-EAS293:2:5:855:1658#0/1    -----------------------------------------------------------------TCAAATGATTTGCACGTCAGTCTGATCACA---
@HWI-EAS293:2:6:905:300#0/1    -----------------------------------------------------------------TCAAATGATTTGCACGTCAGTCTGATCACA---
@HWI-EAS293:2:7:1526:712#0/1    -----------------------------------------------------------------TCAAATGATTTGCACGTCAGTCTGATCACA---
@HWI-EAS293:2:8:364:392#0/1    -----------------------------------------------------------------TCAAATGATTTGCACGTCAGTCTGATCACA---
@HWI-EAS293:2:8:1513:1455#0/1    -----------------------------------------------------------------TCAAATGATTTGCACGTCAGTCTGATCACA---
@HWI-EAS293:2:11:531:562#0/1    -----------------------------------------------------------------TCAAATGATTTGCACGTCAGTCTGATCACA---
@HWI-EAS293:2:13:1600:794#0/1    -----------------------------------------------------------------TCAAATGATTTGCACGTCAGTCTGATCACA---
@HWI-EAS293:2:14:1455:1785#0/1    -----------------------------------------------------------------TCAAATGATTTGCACGTCAGTCTGATCACA---
@HWI-EAS293:2:15:744:648#0/1    -----------------------------------------------------------------TCAAATGATTTGCACGTCAGTCTGATCACA---
@HWI-EAS293:2:18:595:1116#0/1    -----------------------------------------------------------------TCAAATGATTTGCACGTCAGTCTGATCACA---
@HWI-EAS293:2:18:1629:834#0/1    -----------------------------------------------------------------TCAAATGATTTGCACGTCAGTCTGATCACA---
@HWI-EAS293:2:1:1457:582#0/1    ------------------------------------------------------------------CAAATGATTTGCACGTCAGTCTGATCA-----
@HWI-EAS293:2:2:1198:1982#0/1    ------------------------------------------------------------------CAAATGATTTGCACGTCAGTCTGATCACA---
@HWI-EAS293:2:6:1289:235#0/1    ------------------------------------------------------------------CAAATGATTTGCACGTCAGTCTGATCA-----
@HWI-EAS293:2:9:1066:751#0/1    ------------------------------------------------------------------CAAATGATTTGCACGTCAGTCTGATCA-----
@HWI-EAS293:2:9:1077:1754#0/1    ------------------------------------------------------------------CAAATGATTTGCACGTCAGTCTGATCA-----
@HWI-EAS293:2:1:92:2033#0/1    -------------------------------------------------------------------AAATGATTTGCACGTCAGTCTGATCACA---
@HWI-EAS293:2:1:1160:1217#0/1    -------------------------------------------------------------------AAATGATTTGCACGTCAGTCTGATCAC----
@HWI-EAS293:2:1:1191:1702#0/1    -------------------------------------------------------------------AAATGATTTGCACGTCAGTCTGATCAa----
```

```
@HWI-EAS293:2:1:1438:880#0/1     ------------------------------------------------------------------AAATGATTTGCACGTCAGTCTGATCACA---
@HWI-EAS293:2:2:85:281#0/1       ------------------------------------------------------------------AAATGATTTGCACGTCAGTCTGATCACA---
@HWI-EAS293:2:2:1208:636#0/1     ------------------------------------------------------------------AAATGATTTGCACGTCAGTCTGATC------
@HWI-EAS293:2:2:1261:1369#0/1    ------------------------------------------------------------------AAATGATTTGCACGTCAGTCTGATCACA---
@HWI-EAS293:2:2:1266:1044#0/1    ------------------------------------------------------------------AAATGATTTGCACGTCAGTCTGATCAC----
@HWI-EAS293:2:2:1278:1018#0/1    ------------------------------------------------------------------AAATGATTTGCACGTCAGTCTGATCACA---
@HWI-EAS293:2:2:1530:1971#0/1    ------------------------------------------------------------------AAATGATTTGCACGTCAGTCTGATCAC----
@HWI-EAS293:2:3:247:74#0/1       ------------------------------------------------------------------AAATGATTTGCACGTCAGTCTGATCACA---
@HWI-EAS293:2:3:523:83#0/1       ------------------------------------------------------------------AAATGATTTGCACGTCAGTCTGATCAC----
@HWI-EAS293:2:4:7:1685#0/1       ------------------------------------------------------------------AAATGATTTGCACGTCAG-------------
@HWI-EAS293:2:4:27:1509#0/1      ------------------------------------------------------------------AAATGATTTGCACGTCAGTCTGATCACA---
@HWI-EAS293:2:4:32:485#0/1       ------------------------------------------------------------------AAATGATTTGCACGTCAGTCTGATCACA---
@HWI-EAS293:2:4:206:762#0/1      ------------------------------------------------------------------AAATGATTTGCACGTCAGTCTG---------
@HWI-EAS293:2:4:962:1367#0/1     ------------------------------------------------------------------AAATGATTTGCACGTCAGTCTGATCAa----
@HWI-EAS293:2:4:1196:1581#0/1    ------------------------------------------------------------------AAATGATTTGCACGTCAGTCTG---------
@HWI-EAS293:2:4:1209:1713#0/1    ------------------------------------------------------------------AAATGATTTGCACGTCAGTCTGATCACA---
@HWI-EAS293:2:4:1659:1039#0/1    ------------------------------------------------------------------AAATGATTTGCACGTCAGTCTGATCACA---
@HWI-EAS293:2:4:1740:1910#0/1    ------------------------------------------------------------------AAATGATTTGCACGTCAGTCTGATCACA---
@HWI-EAS293:2:5:84:678#0/1       ------------------------------------------------------------------AAATGATTTGCACGTCAGTCTGATCAC----
@HWI-EAS293:2:5:937:1492#0/1     ------------------------------------------------------------------AAATGATTTGCACGTCAGTCTGATCAC----
@HWI-EAS293:2:5:1091:91#0/1      ------------------------------------------------------------------AAATGATTTGCACGTCAGTCTGATCACA---
@HWI-EAS293:2:5:1463:920#0/1     ------------------------------------------------------------------AAATGATTTGCACGTCAGTCTGATCAC----
@HWI-EAS293:2:5:1466:104#0/1     ------------------------------------------------------------------AAATGATTTGCACGTCAGTCTGATCACA---
@HWI-EAS293:2:6:68:1173#0/1      ------------------------------------------------------------------AAATGATTTGCACGTCAGTCTGATCACA---
@HWI-EAS293:2:6:715:463#0/1      ------------------------------------------------------------------AAATGATTTGCACGTCAGTCTGATC------
@HWI-EAS293:2:6:1449:1061#0/1    ------------------------------------------------------------------AAATGATTTGCACGTCAGTCTG---------
@HWI-EAS293:2:7:8:1951#0/1       ------------------------------------------------------------------AAATGATTTGCACGTCAGTCTGA--------
@HWI-EAS293:2:7:315:1546#0/1     ------------------------------------------------------------------AAATGATTTGCACGTCAGTCTGATCACA---
@HWI-EAS293:2:7:518:1956#0/1     ------------------------------------------------------------------AAATGATTTGCACGTCAGTCTGATC------
@HWI-EAS293:2:7:1434:1349#0/1    ------------------------------------------------------------------AAATGATTTGCACGTCAGTCTGATCAC----
@HWI-EAS293:2:8:586:1478#0/1     ------------------------------------------------------------------AAATGATTTGCACGTCAGTCTGATCAC----
@HWI-EAS293:2:8:662:194#0/1      ------------------------------------------------------------------AAATGATTTGCACGTCAGTCTGATCACA---
@HWI-EAS293:2:9:57:1356#0/1      ------------------------------------------------------------------AAATGATTTGCACGTCAGTCTGATCAa----
@HWI-EAS293:2:9:139:1470#0/1     ------------------------------------------------------------------AAATGATTTGCACGTCAGTCTGATCACA---
@HWI-EAS293:2:9:600:387#0/1      ------------------------------------------------------------------AAATGATTTGCACGTCAGTCTGATCACA---
@HWI-EAS293:2:9:725:1068#0/1     ------------------------------------------------------------------AAATGATTTGCACGTCAGTCTGATCACA---
@HWI-EAS293:2:9:788:1655#0/1     ------------------------------------------------------------------AAATGATTTGCACGTCAGTCTG---------
@HWI-EAS293:2:9:1229:1285#0/1    ------------------------------------------------------------------AAATGATTTGCACGTCAGTCTGATCACA---
@HWI-EAS293:2:9:1346:371#0/1     ------------------------------------------------------------------AAATGATTTGCACGTCAGTCTGATCACA---
@HWI-EAS293:2:9:1403:899#0/1     ------------------------------------------------------------------AAATGATTTGCACGTCAGTCTGATCACA---
@HWI-EAS293:2:9:1593:886#0/1     ------------------------------------------------------------------AAATGATTTGCACGTCAGTCTG---------
@HWI-EAS293:2:10:87:206#0/1      ------------------------------------------------------------------AAATGATTTGCACGTCAGTCTGATCACA---
@HWI-EAS293:2:10:673:695#0/1     ------------------------------------------------------------------AAATGATTTGCACGTCAGTCTGATCAC----
@HWI-EAS293:2:10:1018:709#0/1    ------------------------------------------------------------------AAATGATTTGCACGTCAGTCTGATCACA---
@HWI-EAS293:2:10:1056:925#0/1    ------------------------------------------------------------------AAATGATTTGCACGTCAGTCTGATCACA---
@HWI-EAS293:2:10:1108:1625#0/1   ------------------------------------------------------------------AAATGATTTGCACGTCAGTCTGATCAC----
@HWI-EAS293:2:10:1283:192#0/1    ------------------------------------------------------------------AAATGATTTGCACGTCAGTCTGATC------
@HWI-EAS293:2:11:135:1528#0/1    ------------------------------------------------------------------AAATGATTTGCACGTCAGTCTGATCAC----
@HWI-EAS293:2:12:207:574#0/1     ------------------------------------------------------------------AAATGATTTGCACGTCAGTCTGATCACA---
@HWI-EAS293:2:12:773:84#0/1      ------------------------------------------------------------------AAATGATTTGCACGTCAGTCTGATCACA---
@HWI-EAS293:2:12:1065:1916#0/1   ------------------------------------------------------------------AAATGATTTGCACGTCAGTCTGATCACA---
@HWI-EAS293:2:13:535:1481#0/1    ------------------------------------------------------------------AAATGATTTGCACGTCAGTCTGATCAC----
@HWI-EAS293:2:13:548:1104#0/1    ------------------------------------------------------------------AAATGATTTGCACGTCAGTCTG---------
@HWI-EAS293:2:13:1615:783#0/1    ------------------------------------------------------------------AAATGATTTGCACGTCAGTCTGATCACA---
@HWI-EAS293:2:13:1739:1815#0/1   ------------------------------------------------------------------AAATGATTTGCACGTCAGTCTGATCACA---
@HWI-EAS293:2:15:213:1756#0/1    ------------------------------------------------------------------AAATGATTTGCACGTCAGTCTGATCACA---
@HWI-EAS293:2:15:225:1853#0/1    ------------------------------------------------------------------AAATGATTTGCACGTCAGTCTGATCACA---
@HWI-EAS293:2:15:421:1716#0/1    ------------------------------------------------------------------AAATGATTTGCACGTCAGTCTGATCACA---
@HWI-EAS293:2:15:1079:512#0/1    ------------------------------------------------------------------AAATGATTTGCACGTCAGTCTGATCAC----
@HWI-EAS293:2:15:1674:713#0/1    ------------------------------------------------------------------AAATGATTTGCACGTCAGTCTGATCAC----
@HWI-EAS293:2:16:453:1187#0/1    ------------------------------------------------------------------AAATGATTTGCACGTCAGTCTGATC------
@HWI-EAS293:2:16:733:489#0/1     ------------------------------------------------------------------AAATGATTTGCACGTCAGTCTGATC------
@HWI-EAS293:2:16:1690:22#0/1     ------------------------------------------------------------------AAATGATTTGCACGTCAGTCTGATCACA---
@HWI-EAS293:2:16:1699:958#0/1    ------------------------------------------------------------------AAATGATTTGCACGTCAGTCTGATCACA---
@HWI-EAS293:2:16:1701:352#0/1    ------------------------------------------------------------------AAATGATTTGCACGTCAGTCTGATCACA---
@HWI-EAS293:2:17:520:1318#0/1    ------------------------------------------------------------------AAATGATTTGCACGTCAGTCTGATCAC----
@HWI-EAS293:2:17:855:525#0/1     ------------------------------------------------------------------AAATGATTTGCACGTCAGTCTGATCACA---
@HWI-EAS293:2:17:1474:1302#0/1   ------------------------------------------------------------------AAATGATTTGCACGTCAGTCTGATCACA---
@HWI-EAS293:2:18:1035:1369#0/1   ------------------------------------------------------------------AAATGATTTGCACGTCAGTCTG---------
@HWI-EAS293:2:18:1407:936#0/1    ------------------------------------------------------------------AAATGATTTGCACGTCAGTCTGATCAC----
@HWI-EAS293:2:8:1120:218#0/1     --------------------------------------------------------------------ATGATTTGCACGTCAGTCTGATCAC----
@HWI-EAS293:2:13:861:690#0/1     --------------------------------------------------------------------ATGATTTGCACGTCAGTCTGATCAC----
```

rDNA 2350-2550 *in vivo*

```
rDNA 2350-2550 in vivo           caccgcccgtcgctagtaccgattgaatggcttagtgaggcctcaggatctgcttagagaagggggcaactccatctcagagcggagaatttggacaaacttggtcatttagaggaactaaaagtcgtaacaaggtttccgtaggtgaacctgcggaaggatcattaaagaaatttaataattttgaaaatggatttttt
@HWI-EAS293:2:1:35:707#0/1       --------------AGcACCGATTGAATGGC-------------------------------------------------------------------------------------------------------------------------------------------------------------------------
@HWI-EAS293:2:1:65:2021#0/1      -----------------------------------------------------------------------------------------------------------------------------------AAGGTTTCCGTAGGTGAACCTGCGGAAGGATC-------------------------------------
@HWI-EAS293:2:1:16:13#0/1        -----------------------------------------------------------------------------------------------------------------------------------AAGGTTTCCGTAGG--AACCTGCG---------------------------------------------
@HWI-EAS293:2:1:17:1318#0/1      -----------------------------------------------------------------------------------------------------------------------------------AAGGTTTCCGTAGGTGAAC--------------------------------------------------
@HWI-EAS293:2:1:62:915#0/1       -----------------------------------------------------------------------------------------------------------------------------------AAGGTTTCCGTAGGT------------------------------------------------------
@HWI-EAS293:2:1:51:1016#0/1      -----------------------------------------------------------------------------------------------------------------------------------AAGGTTTCCGTAGGT------------------------------------------------------
@HWI-EAS293:2:1:23:451#0/1       ------------------------------------------------------------------------------------------------------------------------------GTAACAAGGTcTCCGT----------------------------------------------------------
@HWI-EAS293:2:1:79:1085#0/1      --------------------------------------------------------------------------------------------------------------AGAGGAACTAAAAGTCGTAAC---------------------------------------------------------------------
@HWI-EAS293:2:1:66:309#0/1       --------------------------------------------------------------------------------------------------------------AGAGGAACTAAAAGTCGTAAC---------------------------------------------------------------------
@HWI-EAS293:2:1:62:763#0/1       --------------------------------------------------------------------------------------------------------------AGAGGAACTAAAAGTCGTAAC---------------------------------------------------------------------
@HWI-EAS293:2:1:57:1698#0/1      --------------------------------------------------------------------------------------------------------------AGAGGAACTAAAAGTCGTAAC---------------------------------------------------------------------
@HWI-EAS293:2:1:44:1697#0/1      --------------------------------------------------------------------------------------------------------------AGAGGAACTAAAAGTCGTAAC---------------------------------------------------------------------
@HWI-EAS293:2:1:41:1999#0/1      --------------------------------------------------------------------------------------------------------------AGAGGAACTAAAAGTCGTAAC---------------------------------------------------------------------
@HWI-EAS293:2:1:40:1180#0/1      --------------------------------------------------------------------------------------------------------------AGAGGAACTAAAAGTCGTAAC---------------------------------------------------------------------
@HWI-EAS293:2:1:39:1939#0/1      ----------------------------------------------------------------------------------------------------------------------TAAAAGTCGTAAC---------------------------------------------------------------------
@HWI-EAS293:2:1:35:760#0/1       --------------------------------------------------------------------------------------------------------------AGAGGAACTAAAAGTCGTAAC---------------------------------------------------------------------
@HWI-EAS293:2:1:33:800#0/1       --------------------------------------------------------------------------------------------------------------AGAGGAACTAAAAGTCGcAAC---------------------------------------------------------------------
@HWI-EAS293:2:1:27:1462#0/1      --------------------------------------------------------------------------------------------------------------AGAGGAACTAAAAGTCGTAAC---------------------------------------------------------------------
@HWI-EAS293:2:1:15:69#0/1        --------------------------------------------------------------------------------------------------------------AGAGGAACTAAAAGTCGcAAC---------------------------------------------------------------------
@HWI-EAS293:2:1:76:540#0/1       -------------------------------------------------------------------AACTCCATCTCAGAGCGGAGAATTTGGACAAACTTGGTCATTTAGAGGAA---AAAG----------------------------------------------------------------------------
@HWI-EAS293:2:1:76:1612#0/1      -------------------------------------------------------------------AACTCCATCTCAGAGCGGAGAATTTGGACAAACTTGGTCATTTAGAGGAA--AAAAG----------------------------------------------------------------------------
@HWI-EAS293:2:1:73:1333#0/1      -------------------------------------------------------------------AACTCCATCTCAGAGCGGAGAATTTGGACAAACTTGGTCATTTAGAGGAA--AAAAG----------------------------------------------------------------------------
@HWI-EAS293:2:1:69:1574#0/1      -------------------------------------------------------------------AACTCCATCTCAGAGCGGAGAATTTGGACAAACTTGGTCATTTAGAGGAA--AAAAG----------------------------------------------------------------------------
@HWI-EAS293:2:1:59:1900#0/1      ---------------------------------------------------------------------------------------AATTTGGACAAACTTGGTCATTTAGAGGAA--AAAAG----------------------------------------------------------------------------
@HWI-EAS293:2:1:38:480#0/1       -------------------------------------------------------------------AACTCCATCTCAGAGCGGAGAATTTGGACAAACTTGGTCATTTAGAGGAA--AAAAG----------------------------------------------------------------------------
@HWI-EAS293:2:1:35:1225#0/1      -------------------------------------------------------------------AACTCCATCTCAGAGCGGAGAATTTGGACAAACTTGGTCATTTAGAGGAA--AAAAG----------------------------------------------------------------------------
@HWI-EAS293:2:1:32:570#0/1       -------------------------------------------------------------------AACTCCATCTCAGAGCGGAGAATTTGGACAAACTTGGTCATTTAGAGGAA--AAAAG----------------------------------------------------------------------------
@HWI-EAS293:2:1:69:285#0/1       ----------------------------------------------------------------------------------------------------TTGGTCATTTAGAGGAAC----------------------------------------------------------------------------------
@HWI-EAS293:2:1:66:1237#0/1      ------------------------------------------------------------------------------------------------AAACTTGGTCATTTAGAGGAAC----------------------------------------------------------------------------------
@HWI-EAS293:2:1:79:966#0/1       -----------------------------------------------------------------------------------GGAGAATTTGGAC--------------------------------------------------------------------------------------------------------
@HWI-EAS293:2:1:79:1189#0/1      -------------------------------------------------------AGAGAAGGGGGCAACTCCATCTCAGAGCGGAGAATT-GGAC--------------------------------------------------------------------------------------------------------
@HWI-EAS293:2:1:76:1850#0/1      -------------------------------------------------------------------AACTCCATCTCAGAGCGGAGAATT-GGAC--------------------------------------------------------------------------------------------------------
@HWI-EAS293:2:1:75:1430#0/1      -----------------------------------------------------------------------------------GGAGAATTTGGAC--------------------------------------------------------------------------------------------------------
@HWI-EAS293:2:1:71:1309#0/1      -----------------------------------------------------------------------------------GGAGAATTTGGAC--------------------------------------------------------------------------------------------------------
@HWI-EAS293:2:1:70:964#0/1       ----------------------------------------------------------------------------------CGGAGAATTTGGAC--------------------------------------------------------------------------------------------------------
@HWI-EAS293:2:1:70:1815#0/1      -------------------------------------------------------------------AACTCCATCTCAGAGCGGAGAATT-GGAC--------------------------------------------------------------------------------------------------------
@HWI-EAS293:2:1:70:1668#0/1      ----------------------------------------------------------------------------------CGGAGAATTTGGAC--------------------------------------------------------------------------------------------------------
@HWI-EAS293:2:1:6:341#0/1        ------------------------------------------------------------------------------AGAGCGGAGAATTTGGAC--------------------------------------------------------------------------------------------------------
@HWI-EAS293:2:1:68:297#0/1       ------------------------------------------------------------------------------AGAGCGGAGAATTTGGAC--------------------------------------------------------------------------------------------------------
@HWI-EAS293:2:1:63:1185#0/1      --------------------------------------------------------------------------------AGCGGAGAATTTGGAC--------------------------------------------------------------------------------------------------------
@HWI-EAS293:2:1:62:1674#0/1      -----------------------------------------------------------------------------------GGAGAATTTGGAC--------------------------------------------------------------------------------------------------------
@HWI-EAS293:2:1:59:1628#0/1      -----------------------------------------------------------------------------------GGAGAATTTGGAC--------------------------------------------------------------------------------------------------------
@HWI-EAS293:2:1:58:1548#0/1      -------------------------------------------------------------------AACTCCATCTCAGAGCGGAGAATT-GGAC--------------------------------------------------------------------------------------------------------
@HWI-EAS293:2:1:58:1185#0/1      -------------------------------------------------------------------------ATCTCAGAGCGGAGAATTTGGAC--------------------------------------------------------------------------------------------------------
@HWI-EAS293:2:1:56:276#0/1       ------------------------------------------------------------------------------AGAGCGGAGAATTcGGAC--------------------------------------------------------------------------------------------------------
@HWI-EAS293:2:1:56:1067#0/1      -------------------------------------------------------------------AACTCCATCTCAGAGCGGAGAATT-GGAC--------------------------------------------------------------------------------------------------------
@HWI-EAS293:2:1:52:314#0/1       -------------------------------------------------------------------AACTCCATCTCAGAGCGGAGAATT-GGAC--------------------------------------------------------------------------------------------------------
@HWI-EAS293:2:1:49:123#0/1       ------------------------------------------------------------------------------AGAGCGGAGAATTTGGAC--------------------------------------------------------------------------------------------------------
@HWI-EAS293:2:1:45:539#0/1       -----------------------------------------------------------------------------------GGAGAATTTGGAC--------------------------------------------------------------------------------------------------------
@HWI-EAS293:2:1:44:1028#0/1      -----------------------------------------------------------------------------------GGAGAATTTGGAC--------------------------------------------------------------------------------------------------------
@HWI-EAS293:2:1:43:602#0/1       -----------------------------------------------------------------------------------GGAGAATTTGGAC--------------------------------------------------------------------------------------------------------
@HWI-EAS293:2:1:38:764#0/1       --------------------------------------------------------------------------------AGCGGAGAATTTGGAC--------------------------------------------------------------------------------------------------------
@HWI-EAS293:2:1:37:774#0/1       -----------------------------------------------------------------------------------GGAGAATTTGGAC--------------------------------------------------------------------------------------------------------
@HWI-EAS293:2:1:30:1698#0/1      -------------------------------------------------------------------AACTCCATCTCAGAGCGGAGAATT-GGAC--------------------------------------------------------------------------------------------------------
@HWI-EAS293:2:1:23:301#0/1       ------------------------------------------------------------------------------AGAGCGGAGAATTTGGAC--------------------------------------------------------------------------------------------------------
@HWI-EAS293:2:1:23:1418#0/1      -------------------------------------------------------------------------ATCTCAGAGCGGAGAATT-GGAC--------------------------------------------------------------------------------------------------------
@HWI-EAS293:2:1:20:1612#0/1      -----------------------------------------------------------------------------------GGAGAATTTGGAC--------------------------------------------------------------------------------------------------------
@HWI-EAS293:2:1:14:440#0/1       -------------------------------------------------------------------AACTCCATCTCAGAGCGGAGAATTTGGAC--------------------------------------------------------------------------------------------------------
@HWI-EAS293:2:1:33:756#0/1       ------------------------------------GAGGCCTCAGGATCTGCT-AGAGAAG------------------------------------------------------------------------------------------------------------------------------------------
```

rDNA 4400-4500 *in vivo*

```
rDNA 4400-4500 in vivo            gggccatttttggtaagcagaactggcgatgcgggatgaaccgaacgtagagttaaggtgccggaatacacgctcatcagacaccacaaaaggtgttagt
@HWI-EAS293:2:1:28:1482#0/1       ------------------------------------------------------AAGGTGCCGGAATACACGCTCAgAC---------------------
@HWI-EAS293:2:1:38:613#0/1        ------------------------GGCGATGCGGGATGAACCGAACGT----------------------------------------------------
@HWI-EAS293:2:1:8:776#0/1         ---------------------------GATGCGGGATGAAC-----------------------------------------------------------
@HWI-EAS293:2:1:81:483#0/1        ---------------------------GATGCGGGATGAAC-----------------------------------------------------------
@HWI-EAS293:2:1:79:862#0/1        --------------------------CGATGCGGGATGAAC-----------------------------------------------------------
@HWI-EAS293:2:1:79:296#0/1        ---------------------------GATGCGGGATGAAC-----------------------------------------------------------
@HWI-EAS293:2:1:77:748#0/1        ---------------------------GATGCGGGATGAAC-----------------------------------------------------------
@HWI-EAS293:2:1:77:29#0/1         ---------------------------GATGCGGGATGAAC-----------------------------------------------------------
@HWI-EAS293:2:1:76:1552#0/1       ---------------------------GATGCGGGATGAAC-----------------------------------------------------------
@HWI-EAS293:2:1:76:1068#0/1       ---------------------------GATGCGGGATGAAC-----------------------------------------------------------
@HWI-EAS293:2:1:75:90#0/1         ---------------------------GATGCGGGATGAAC-----------------------------------------------------------
@HWI-EAS293:2:1:75:1957#0/1       ---------------------------GATGCGGGATGAAC-----------------------------------------------------------
@HWI-EAS293:2:1:75:1713#0/1       ---------------------------GATGCGGGATGAAC-----------------------------------------------------------
@HWI-EAS293:2:1:74:1354#0/1       ---------------------------GATGCGGGATGAAC-----------------------------------------------------------
@HWI-EAS293:2:1:72:1965#0/1       ---------------------------GATGCGGGATGAAC-----------------------------------------------------------
@HWI-EAS293:2:1:72:1498#0/1       ---------------------------GATGCGGGATGAAC-----------------------------------------------------------
@HWI-EAS293:2:1:69:1921#0/1       ---------------------------GATGCGGGATGAAC-----------------------------------------------------------
@HWI-EAS293:2:1:68:984#0/1        ---------------------------GATGCGGGATGAAC-----------------------------------------------------------
@HWI-EAS293:2:1:68:2040#0/1       ---------------------------GATGCGGGATGAAC-----------------------------------------------------------
@HWI-EAS293:2:1:68:1908#0/1       ---------------------------GATGCGGGATGAAC-----------------------------------------------------------
@HWI-EAS293:2:1:68:1878#0/1       ---------------------------GATGCGGGATGAAC-----------------------------------------------------------
@HWI-EAS293:2:1:68:1303#0/1       ----------------------------ATGCGGGATGAAC-----------------------------------------------------------
@HWI-EAS293:2:1:67:618#0/1        ---------------------------GATGCGGGATGAAC-----------------------------------------------------------
@HWI-EAS293:2:1:66:1137#0/1       ---------------------------GATGCGGGATGAAC-----------------------------------------------------------
@HWI-EAS293:2:1:65:2001#0/1       ---------------------------GATGCGGGATGAAC-----------------------------------------------------------
@HWI-EAS293:2:1:65:1941#0/1       ----------------------------ATGCGGGATGAAC-----------------------------------------------------------
@HWI-EAS293:2:1:64:520#0/1        ---------------------------GATGCGGGATGAAC-----------------------------------------------------------
@HWI-EAS293:2:1:64:1436#0/1       ---------------------------GATGCGGGATGAAC-----------------------------------------------------------
@HWI-EAS293:2:1:63:849#0/1        ----------------------------ATGCGGGATGAAC-----------------------------------------------------------
@HWI-EAS293:2:1:62:833#0/1        ---------------------------GATGCGGGATGAAC-----------------------------------------------------------
@HWI-EAS293:2:1:61:929#0/1        ---------------------------GATGCGGGATGAAC-----------------------------------------------------------
@HWI-EAS293:2:1:59:1039#0/1       ---------------------------GATGCGGGATGAAC-----------------------------------------------------------
@HWI-EAS293:2:1:57:1281#0/1       ---------------------------GATGCGGGATGAAC-----------------------------------------------------------
@HWI-EAS293:2:1:56:1644#0/1       ---------------------------GATGCGGGATGAAC-----------------------------------------------------------
@HWI-EAS293:2:1:55:667#0/1        ---------------------------GATGCGGGATGAAC-----------------------------------------------------------
@HWI-EAS293:2:1:54:491#0/1        ----------------------------ATGCGGGATGAAC-----------------------------------------------------------
@HWI-EAS293:2:1:53:770#0/1        ---------------------------GATGCGGGATGAAC-----------------------------------------------------------
@HWI-EAS293:2:1:52:803#0/1        ---------------------------GATGCGGGATGAAC-----------------------------------------------------------
@HWI-EAS293:2:1:52:695#0/1        ---------------------------GATGCGGGATGAAC-----------------------------------------------------------
@HWI-EAS293:2:1:49:545#0/1        --------------------------CGATGCGGGATGAAC-----------------------------------------------------------
@HWI-EAS293:2:1:48:1830#0/1       ---------------------------GATGCGGGATGAAC-----------------------------------------------------------
@HWI-EAS293:2:1:48:1287#0/1       --------------------------CGATGCGGGATGAAC-----------------------------------------------------------
@HWI-EAS293:2:1:47:578#0/1        --------------------------CGATGCGGGATGAAC-----------------------------------------------------------
@HWI-EAS293:2:1:42:429#0/1        ---------------------------GATGCGGGATGAAC-----------------------------------------------------------
@HWI-EAS293:2:1:42:1332#0/1       --------------------------CGATGCGGGATGAAC-----------------------------------------------------------
@HWI-EAS293:2:1:42:1225#0/1       ----------------------------ATGCGGGATGAAC-----------------------------------------------------------
@HWI-EAS293:2:1:41:1537#0/1       ----------------------------ATGCGGGATGAAC-----------------------------------------------------------
@HWI-EAS293:2:1:40:563#0/1        ---------------------------GATGCGGGATGAAC-----------------------------------------------------------
@HWI-EAS293:2:1:37:667#0/1        ---------------------------GATGCGGGATGAAC-----------------------------------------------------------
@HWI-EAS293:2:1:36:1827#0/1       ---------------------------GATGCGGGATGAAC-----------------------------------------------------------
@HWI-EAS293:2:1:34:798#0/1        ---------------------------GATGCGGGATGAAC-----------------------------------------------------------
@HWI-EAS293:2:1:34:1513#0/1       ---------------------------GATGCGGGATGAAC-----------------------------------------------------------
@HWI-EAS293:2:1:34:1003#0/1       ---------------------------GATGCGGGATGAAC-----------------------------------------------------------
@HWI-EAS293:2:1:32:849#0/1        ---------------------------GATGCGGGATGAAC-----------------------------------------------------------
@HWI-EAS293:2:1:32:451#0/1        ---------------------------GATGCGGGATGAAC-----------------------------------------------------------
@HWI-EAS293:2:1:31:88#0/1         --------------------------CGATGCGGGATGAAC-----------------------------------------------------------
@HWI-EAS293:2:1:30:665#0/1        ---------------------------GATGCGGGATGAAC-----------------------------------------------------------
@HWI-EAS293:2:1:30:1859#0/1       ----------------------------ATGCGGGATGAAC-----------------------------------------------------------
@HWI-EAS293:2:1:29:423#0/1        ---------------------------GATGCGGGATGAAC-----------------------------------------------------------
@HWI-EAS293:2:1:27:300#0/1        ---------------------------GATGCGGGATGAAC-----------------------------------------------------------
@HWI-EAS293:2:1:26:2026#0/1       ---------------------------GATGCGGGATGAAC-----------------------------------------------------------
@HWI-EAS293:2:1:26:1350#0/1       ----------------------------ATGCGGGATGAAC-----------------------------------------------------------
@HWI-EAS293:2:1:26:1067#0/1       ---------------------------GATGCGGGATGAAC-----------------------------------------------------------
@HWI-EAS293:2:1:25:336#0/1        ---------------------------GATGCGGGATGAAC-----------------------------------------------------------
@HWI-EAS293:2:1:24:1642#0/1       ---------------------------GATGCGGGATGAAC-----------------------------------------------------------
@HWI-EAS293:2:1:24:1589#0/1       ----------------------------ATGCGGGATGAAC-----------------------------------------------------------
@HWI-EAS293:2:1:22:1159#0/1       ---------------------------GATGCGGGATGAAC-----------------------------------------------------------
@HWI-EAS293:2:1:19:957#0/1        ---------------------------GATGCGGGATGAAC-----------------------------------------------------------
@HWI-EAS293:2:1:19:1434#0/1       ---------------------------GATGCGGGATGAAC-----------------------------------------------------------
@HWI-EAS293:2:1:17:1139#0/1       ----------------------------ATGCGGGATGAAC-----------------------------------------------------------
@HWI-EAS293:2:1:16:528#0/1        ---------------------------GATGCGGGATGAAC-----------------------------------------------------------
@HWI-EAS293:2:1:14:141#0/1        ---------------------------GATGCGGGATGAAC-----------------------------------------------------------
@HWI-EAS293:2:1:12:500#0/1        ---------------------------GATGCGGGATGAAC-----------------------------------------------------------
@HWI-EAS293:2:1:12:1986#0/1       ---------------------------GATGCGGGATGAAC-----------------------------------------------------------
@HWI-EAS293:2:1:12:1625#0/1       ------------------------GGCGATGCGGGATGAAC-----------------------------------------------------------
@HWI-EAS293:2:1:11:795#0/1        ---------------------------GATGCGGGATGAAC-----------------------------------------------------------
@HWI-EAS293:2:1:11:1726#0/1       ----------------------------ATGCGGGATGAAC-----------------------------------------------------------
```

rDNA 3600-3800 *in vitro*

```
rDNA 3600-3800 in vitro         ctaaagctaaatattggcgagagaccgatagcgaacaagtacagtgatggaaagatgaaaagaactttgaaaagagagtgaaaaagtacgtgaaattgttgaaagggaagggcatttgatcagacatggtgttttgtgccctctgctccttgtgggtaggggaatctcgcatttcactgggccagcatcagttttggtggc
@HWI-EAS293:3:1:49:712#0/1      --------------------------------------------------------GAAAAGAACTT-GAAAAGAGAGTGAAAAAGT------------------------------------------------------------------------------------------------------------------
@HWI-EAS293:3:1:17:1239#0/1     --------------------------------------------------------GAAAAGAACTT-GAAAAGAGAGTGAAAAAGT------------------------------------------------------------------------------------------------------------------
@HWI-EAS293:3:1:14:944#0/1      ------------------------------------------------GGAAAGATGAAAAGAACTTTGAAAAGAGAGTGAAAA---------------------------------------------------------------------------------------------------------------------
@HWI-EAS293:3:1:43:425#0/1      ------------------------------------------------GGAAAGATGAAAAGAACTTTGAAAAGAGAGT--------------------------------------------------------------------------------------------------------------------------
@HWI-EAS293:3:1:41:202#0/1      ------------------------------------------AGTGATGGAAAGATGAAAAGAACTTTGAAAAGAGAGT--------------------------------------------------------------------------------------------------------------------------
@HWI-EAS293:3:1:40:1236#0/1     --------------------------------------------------AAAGATGAAAAGAACTTTGAAAAGAGAGT--------------------------------------------------------------------------------------------------------------------------
@HWI-EAS293:3:1:38:1141#0/1     ------------------------------------------AGTGATGGAAAGATGAAAAGAACTTTGAAAAGAGAGT--------------------------------------------------------------------------------------------------------------------------
@HWI-EAS293:3:1:24:458#0/1      ------------------------------------------AGTGATGGAAAGATGAAAAGAACTTTGAAAAGAGAGT--------------------------------------------------------------------------------------------------------------------------
@HWI-EAS293:3:1:48:1631#0/1     ------------------------------------------AGTGATGGAAAGATGAAAAGAACTTTGAAAAGAGAG---------------------------------------------------------------------------------------------------------------------------
@HWI-EAS293:3:1:20:497#0/1      ---------------------------------------------GATGGAAAGATGAAAAGAACTTcGAAAAGAGAG---------------------------------------------------------------------------------------------------------------------------
@HWI-EAS293:3:1:16:288#0/1      ------------------------------------------------GGAAAGATGAAAAGAACTTTGAAAAGAGAG---------------------------------------------------------------------------------------------------------------------------
@HWI-EAS293:3:1:50:693#0/1      ------------------------------------------AGTGATGGAAAGATGAAAAGAACTTTGAAAAG-------------------------------------------------------------------------------------------------------------------------------
@HWI-EAS293:3:1:29:265#0/1      ---------------------------------------------GATGGAAAGATGAAAAGAACTT-GAAAA--------------------------------------------------------------------------------------------------------------------------------
@HWI-EAS293:3:1:16:561#0/1      ------------------------------------------------aGAAAGATGAAAAGAACTTT-------------------------------------------------------------------------------------------------------------------------------------
@HWI-EAS293:3:1:30:524#0/1      ---------------------------------------------GATGGAAAGATGAAAAGAACT---------------------------------------------------------------------------------------------------------------------------------------
@HWI-EAS293:3:1:8:684#0/1       ------------------------------------------AGTGATGGAAAGATGAAAAGAAC----------------------------------------------------------------------------------------------------------------------------------------
@HWI-EAS293:3:1:8:662#0/1       ------------------------------------------AGTGtTGGAAAGATGAAAAGAAC----------------------------------------------------------------------------------------------------------------------------------------
@HWI-EAS293:3:1:50:1968#0/1     ------------------------------------------------GGAAAGATGAAAAGAAC----------------------------------------------------------------------------------------------------------------------------------------
@HWI-EAS293:3:1:49:353#0/1      ------------------------------------------AGTGATGGAAAGATGAAAAGAAC----------------------------------------------------------------------------------------------------------------------------------------
@HWI-EAS293:3:1:48:1208#0/1     ------------------------------------------AGTGATGGAAAGATGAAAAGAAC----------------------------------------------------------------------------------------------------------------------------------------
@HWI-EAS293:3:1:46:980#0/1      ------------------------------------------AGTGATGGAAAGATGAAAAGAAC----------------------------------------------------------------------------------------------------------------------------------------
@HWI-EAS293:3:1:45:1258#0/1     ------------------------------------------------GGAAAGATGAAAAGAAC----------------------------------------------------------------------------------------------------------------------------------------
@HWI-EAS293:3:1:44:1471#0/1     ------------------------------------------------GGAAAGATGAAAAGAAC----------------------------------------------------------------------------------------------------------------------------------------
@HWI-EAS293:3:1:41:636#0/1      ----------------------------------------------ATGGAAAGATGAAAAGAAC----------------------------------------------------------------------------------------------------------------------------------------
@HWI-EAS293:3:1:38:1651#0/1     ------------------------------------------AGTGATGGAA-GATGAAAAGAAC----------------------------------------------------------------------------------------------------------------------------------------
@HWI-EAS293:3:1:34:360#0/1      ------------------------------------------AGTGATGGAAAGATGAAAAGAAC----------------------------------------------------------------------------------------------------------------------------------------
@HWI-EAS293:3:1:34:109#0/1      ------------------------------------------AGTGATGGAAAGATGAAAAGAAC----------------------------------------------------------------------------------------------------------------------------------------
@HWI-EAS293:3:1:34:1061#0/1     ------------------------------------------------GGAAAGATGAAAAGAAC----------------------------------------------------------------------------------------------------------------------------------------
@HWI-EAS293:3:1:31:1146#0/1     ------------------------------------------AGTGATGGAAAGATGAAAAGAAC----------------------------------------------------------------------------------------------------------------------------------------
@HWI-EAS293:3:1:31:1122#0/1     ------------------------------------------AGTGATGGAAAGATGAAAAGAAC----------------------------------------------------------------------------------------------------------------------------------------
@HWI-EAS293:3:1:30:687#0/1      ------------------------------------------AGTGATGGAAAGATGAAAAGAAC----------------------------------------------------------------------------------------------------------------------------------------
@HWI-EAS293:3:1:30:1957#0/1     ---------------------------------------------GATGGAAAGATGAAAAaAAC----------------------------------------------------------------------------------------------------------------------------------------
@HWI-EAS293:3:1:27:141#0/1      ---------------------------------------------GATGGAAAGATGAAAAGAAC----------------------------------------------------------------------------------------------------------------------------------------
@HWI-EAS293:3:1:25:226#0/1      ---------------------------------------------GATGGAAAGATGAAAAGAAC----------------------------------------------------------------------------------------------------------------------------------------
@HWI-EAS293:3:1:24:53#0/1       ------------------------------------------AGTGATGGAAAGATGAAAAGAAC----------------------------------------------------------------------------------------------------------------------------------------
@HWI-EAS293:3:1:24:1863#0/1     ------------------------------------------AGTGATGGAAAGATGAAAAGAAC----------------------------------------------------------------------------------------------------------------------------------------
@HWI-EAS293:3:1:21:1092#0/1     ------------------------------------------AGTGATGGAAAGATGAAAAGAAC----------------------------------------------------------------------------------------------------------------------------------------
@HWI-EAS293:3:1:20:808#0/1      ------------------------------------------AGTGATGGAAAGATGAAAAGAAC----------------------------------------------------------------------------------------------------------------------------------------
@HWI-EAS293:3:1:20:1204#0/1     ------------------------------------------AGTGATGGAAAGATGAAAAGAAC----------------------------------------------------------------------------------------------------------------------------------------
@HWI-EAS293:3:1:19:1738#0/1     ------------------------------------------AGTGATGGAAAGATGAAAAGAAC----------------------------------------------------------------------------------------------------------------------------------------
@HWI-EAS293:3:1:15:508#0/1      ------------------------------------------AGTGATGGAAAGATGAAAAGAAC----------------------------------------------------------------------------------------------------------------------------------------
@HWI-EAS293:3:1:12:1307#0/1     ------------------------------------------AGTGATGGAAAGATGAAAAGAAC----------------------------------------------------------------------------------------------------------------------------------------
@HWI-EAS293:3:1:11:1076#0/1     ------------------------------------------AGTGATGGAAAGATGAAAAGAAC----------------------------------------------------------------------------------------------------------------------------------------
@HWI-EAS293:3:1:43:841#0/1      -------------------------------------------GTGATGGAAAGAT-------------------------------------------------------------------------------------------------------------------------------------------------
@HWI-EAS293:3:1:31:1288#0/1     ------------------------------------------AGTGATGGAAAGAT-------------------------------------------------------------------------------------------------------------------------------------------------
@HWI-EAS293:3:1:27:1402#0/1     -------------------------CGATAGCGAACAAGcAC---------------------------------------------------------------------------------------------------------------------------------------------------------------
@HWI-EAS293:3:1:48:1786#0/1     ------------ATTGGCGAGAGACCGATAGtGAAC---------------------------------------------------------------------------------------------------------------------------------------------------------------------
@HWI-EAS293:3:1:42:2027#0/1     ------------------GAGAGACCGATAGCGAAC---------------------------------------------------------------------------------------------------------------------------------------------------------------------
@HWI-EAS293:3:1:22:1830#0/1     ------------ATTGGCGAGAGACCGATAGCGAAC---------------------------------------------------------------------------------------------------------------------------------------------------------------------
@HWI-EAS293:3:1:10:329#0/1      -----------------------ACCGATAGCGAAC---------------------------------------------------------------------------------------------------------------------------------------------------------------------
@HWI-EAS293:3:1:19:233#0/1      ---------------------------------------------------------------------------------------ACGTGAAATTGTTGAAAGGGAAGGGCATTTGATC--------------------------------------------------------------------------------
@HWI-EAS293:3:1:39:51#0/1       ------------------------------------------------------------------------------------------------TGTTGAAAGGGAAGGGC----------------------------------------------------------------------------------------
@HWI-EAS293:3:1:27:1987#0/1     ---------------------------------------------------------------------------------------ACGTGAAATTGTTGAAAGGGAAGGGC----------------------------------------------------------------------------------------
@HWI-EAS293:3:1:22:139#0/1      ---------------------------------------------------------------------------------------ACGTGAAATTGTTGAAAGGGAAGGGC----------------------------------------------------------------------------------------
@HWI-EAS293:3:1:18:937#0/1      ---------------------------------------------------------------------------------------ACGTGAAATTGTTGAAAGGGAAGGGC----------------------------------------------------------------------------------------
@HWI-EAS293:3:1:46:102#0/1      --------------------------------------------------------------------GAAAAGAGAGTGAAAAAGgACGTGAAATTGTTGAAAGGGAAG-------------------------------------------------------------------------------------------
@HWI-EAS293:3:1:33:112#0/1      -------------------------------------------------------------------------------GAAAAAGTACGTGAAATTGTTGAAAGGGAAG-------------------------------------------------------------------------------------------
@HWI-EAS293:3:1:38:652#0/1      -------------------------------------------------------------------------------------------GAAATTGTTGAAAGGG----------------------------------------------------------------------------------------------
```

rDNA 3900-4100 *in vitro*

```
rDNA 3900-4100 in vitro           tggcataatggttatatgccgcccgtcttgaaacacggaccaaggagtctaacgtctatgcgagtgtttgggtgtaaaacccatacgcgtaatgaaagtgaacgtaggttggggcctcgcaagaggtgcacaatcgaccgatcctgatgtcttcggatggatttgagtaagagcatagctgttgggacccgaaagatggtg
@HWI-EAS293:3:1:8:849#0/1         ---------------ATGCCGCCCGTCTTGAAAC-----------------------------------------------------------------------------------------------------------------------------------------------------------------------
@HWI-EAS293:3:1:16:1286#0/1       --------------------------------------------------------------------------------------------------------------------------------------------------------------------GAGTAAGAGCATAGCTGTTGGGAC-------------
@HWI-EAS293:3:1:10:1665#0/1       ------------------------------------------------------------------------------------------------------------------------------------------------------CTTCGGATGGATT-GAGTAAGAGC---------------------------
@HWI-EAS293:3:1:9:1777#0/1        ------------------------------------------------------------------------------------------------------------------------------------------------------CTTCGGATGGATTTGAGT---------------------------------
@HWI-EAS293:3:1:7:340#0/1         -------------------------------------------------------------------------------------------------------------------------------------------------------TTCGGATGGATTTGAGT---------------------------------
@HWI-EAS293:3:1:37:1756#0/1       -----------------------------------------------------------------------------------------------------------------------------------------------------------GATGGATTTGAGT---------------------------------
@HWI-EAS293:3:1:36:1246#0/1       ---------------------------------------------------------------------------------------------------------------------------------------------------------CGGATGGATTTGAGT---------------------------------
@HWI-EAS293:3:1:31:710#0/1        ------------------------------------------------------------------------------------------------------------------------------------------------------CTTCGGATGGATTTGAGT---------------------------------
@HWI-EAS293:3:1:20:1517#0/1       ------------------------------------------------------------------------------------------------------------------------------------------------------CTTCGGATGGATTcGAG----------------------------------
@HWI-EAS293:3:1:30:384#0/1        -------------------------------------------------------------------------------------------------------GTAGGTTGGGGCCTCGC---------------------------------------------------------------------------------
@HWI-EAS293:3:1:43:1323#0/1       ---------------------------------------------------------------------------------------------GAAAGTGAACGTAGGTTGGGGC--------------------------------------------------------------------------------------
@HWI-EAS293:3:1:34:922#0/1        ------------------------------------------------------------------------------------------AATGAAAGTGAACGTAGGT--------------------------------------------------------------------------------------------
@HWI-EAS293:3:1:9:489#0/1         ------------------------------------------------------------------------------------ACGCGTAATGAAAGTGAAC--------------------------------------------------------------------------------------------------
@HWI-EAS293:3:1:9:1850#0/1        ------------------------------------------------------------------------------------ACGCGTAATGAAAGTGAAC--------------------------------------------------------------------------------------------------
@HWI-EAS293:3:1:8:880#0/1         ----------------------------------------------------------------------------------------GTAAcGAAAGTGAAC--------------------------------------------------------------------------------------------------
@HWI-EAS293:3:1:8:800#0/1         ------------------------------------------------------------------------------------ACGCGTAATGAAAGTGAAC--------------------------------------------------------------------------------------------------
@HWI-EAS293:3:1:8:1684#0/1        --------------------------------------------------------------------------------------GCGTAATGAAAGTGAAC--------------------------------------------------------------------------------------------------
@HWI-EAS293:3:1:50:663#0/1        ------------------------------------------------------------------------------------ACGCGTAATGAAAGTGAAC--------------------------------------------------------------------------------------------------
@HWI-EAS293:3:1:50:1276#0/1       ------------------------------------------------------------------------------------ACGCGTAATGAAAGTGAAC--------------------------------------------------------------------------------------------------
@HWI-EAS293:3:1:48:1804#0/1       ----------------------------------------------------------------------------------------GTAATGAAAGTGAAC--------------------------------------------------------------------------------------------------
@HWI-EAS293:3:1:46:1433#0/1       ------------------------------------------------------------------------------------ACGCGTAATGAAAGTGAAC--------------------------------------------------------------------------------------------------
@HWI-EAS293:3:1:45:1098#0/1       ---------------------------------------------------------------------------------------CGTAATGAAAGTGAAC--------------------------------------------------------------------------------------------------
@HWI-EAS293:3:1:43:673#0/1        --------------------------------------------------------------------------------------GCGTAATGAAAGTGAAC--------------------------------------------------------------------------------------------------
@HWI-EAS293:3:1:42:1955#0/1       ---------------------------------------------------------------------------------------aGTAATGAAAGTGAAC--------------------------------------------------------------------------------------------------
@HWI-EAS293:3:1:42:1219#0/1       ------------------------------------------------------------------------------------ACGCGTAATGAAAGTGAAC--------------------------------------------------------------------------------------------------
@HWI-EAS293:3:1:41:520#0/1        --------------------------------------------------------------------------------------GCGTAATGAAAGTGAAC--------------------------------------------------------------------------------------------------
@HWI-EAS293:3:1:41:447#0/1        ----------------------------------------------------------------------------------ATACGCGTAATGAAAGTGAAC--------------------------------------------------------------------------------------------------
@HWI-EAS293:3:1:41:185#0/1        ------------------------------------------------------------------------------------ACGCGTAATGAAAGTGAAC--------------------------------------------------------------------------------------------------
@HWI-EAS293:3:1:41:1405#0/1       ------------------------------------------------------------------------------------ACGCGTAATaAAAGTGAAC--------------------------------------------------------------------------------------------------
@HWI-EAS293:3:1:40:701#0/1        -----------------------------------------------------------------------------------------TAATGAAAGTGAAC--------------------------------------------------------------------------------------------------
@HWI-EAS293:3:1:40:650#0/1        --------------------------------------------------------------------------------------GCGTAATGAAAGTGAAC--------------------------------------------------------------------------------------------------
@HWI-EAS293:3:1:40:245#0/1        ------------------------------------------------------------------------------------ACGCGTAATGAAAGTGAAC--------------------------------------------------------------------------------------------------
@HWI-EAS293:3:1:40:1526#0/1       ------------------------------------------------------------------------------------ACGCGTAATGAAAGTGAAC--------------------------------------------------------------------------------------------------
@HWI-EAS293:3:1:40:1204#0/1       --------------------------------------------------------------------------------------GCGTAATGAAAGTGAAC--------------------------------------------------------------------------------------------------
@HWI-EAS293:3:1:39:1515#0/1       ----------------------------------------------------------------------------------------GTAATGAAAGTGAAC--------------------------------------------------------------------------------------------------
@HWI-EAS293:3:1:38:1606#0/1       ------------------------------------------------------------------------------------ACGCGTAATGAAAGTGAAC--------------------------------------------------------------------------------------------------
@HWI-EAS293:3:1:35:927#0/1        ------------------------------------------------------------------------------------ACGCGTAATGAAAGTGAAC--------------------------------------------------------------------------------------------------
@HWI-EAS293:3:1:35:622#0/1        ------------------------------------------------------------------------------------ACGCGTAATGAAAGTGAAC--------------------------------------------------------------------------------------------------
@HWI-EAS293:3:1:35:1921#0/1       ------------------------------------------------------------------------------------ACGCGTAAcGAAAGTGAAC--------------------------------------------------------------------------------------------------
@HWI-EAS293:3:1:33:2043#0/1       ----------------------------------------------------------------------------------------GTAATGAAAGTGAAC--------------------------------------------------------------------------------------------------
@HWI-EAS293:3:1:32:893#0/1        ----------------------------------------------------------------------------------------GTAATGAAAGTGAAC--------------------------------------------------------------------------------------------------
@HWI-EAS293:3:1:32:631#0/1        ----------------------------------------------------------------------------------------GTAATGAAAGTGAAC--------------------------------------------------------------------------------------------------
@HWI-EAS293:3:1:31:812#0/1        --------------------------------------------------------------------------------------GCGTAATGAAAGTGAAC--------------------------------------------------------------------------------------------------
@HWI-EAS293:3:1:30:1458#0/1       ----------------------------------------------------------------------------------------GTAATaAAAGTGAAC--------------------------------------------------------------------------------------------------
@HWI-EAS293:3:1:26:1021#0/1       ------------------------------------------------------------------------------------ACGCGTAATGAAAGTGAAC--------------------------------------------------------------------------------------------------
@HWI-EAS293:3:1:25:670#0/1        --------------------------------------------------------------------------------------GCGTAATGAAAGTGAAC--------------------------------------------------------------------------------------------------
@HWI-EAS293:3:1:24:79#0/1         ------------------------------------------------------------------------------------ACGCGTAATGAAAGTGAAC--------------------------------------------------------------------------------------------------
@HWI-EAS293:3:1:24:646#0/1        ------------------------------------------------------------------------------------ACGCGTAATGAAAGTGAAC--------------------------------------------------------------------------------------------------
@HWI-EAS293:3:1:23:1022#0/1       ----------------------------------------------------------------------------------------GTAATGAAAGTGAAC--------------------------------------------------------------------------------------------------
@HWI-EAS293:3:1:20:212#0/1        ------------------------------------------------------------------------------------ACGCGTAATGAAAGTGAAC--------------------------------------------------------------------------------------------------
@HWI-EAS293:3:1:20:1797#0/1       ----------------------------------------------------------------------------------ATACGCGTAATGAAAGTGAAC--------------------------------------------------------------------------------------------------
@HWI-EAS293:3:1:18:51#0/1         ----------------------------------------------------------------------------------ATACGCGTAATGAAAGTGAAC--------------------------------------------------------------------------------------------------
@HWI-EAS293:3:1:18:302#0/1        -----------------------------------------------------------------------------------TACGCGTAATGAAAGTGAAC--------------------------------------------------------------------------------------------------
@HWI-EAS293:3:1:17:520#0/1        ---------------------------------------------------------------------------------------CGTAATGAAAGTGAAC--------------------------------------------------------------------------------------------------
@HWI-EAS293:3:1:17:1037#0/1       ------------------------------------------------------------------------------------ACGCGTAATGAAAGTGAAC--------------------------------------------------------------------------------------------------
@HWI-EAS293:3:1:16:1196#0/1       ----------------------------------------------------------------------------------------GTAATGAAAGcGAAC--------------------------------------------------------------------------------------------------
@HWI-EAS293:3:1:14:1237#0/1       ------------------------------------------------------------------------------------ACGCGTAATGAAAGcGAAC--------------------------------------------------------------------------------------------------
@HWI-EAS293:3:1:12:973#0/1        ----------------------------------------------------------------------------------------GTAATGAAAGTGAAC--------------------------------------------------------------------------------------------------
@HWI-EAS293:3:1:12:1534#0/1       ----------------------------------------------------------------------------------------GTAATGAAAGTGAAC--------------------------------------------------------------------------------------------------
@HWI-EAS293:3:1:12:1469#0/1       ----------------------------------------------------------------------------------------GTAATGAAAGTGAAC--------------------------------------------------------------------------------------------------
@HWI-EAS293:3:1:11:81#0/1         ----------------------------------------------------------------------------------------GTAATGtAAGTGAAC--------------------------------------------------------------------------------------------------
@HWI-EAS293:3:1:10:955#0/1        ------------------------------------------------------------------------------------ACGCGTAATGAAAGTGAAC--------------------------------------------------------------------------------------------------
@HWI-EAS293:3:1:10:771#0/1        --------------------------------------------------------------------------------------GCGTAATGAAAGTGAAC--------------------------------------------------------------------------------------------------
```

rDNA 5900-6000 *in vitro*

```
rDNA 5900-6000 in vitro           gctggggcggcacatctgttaaacgataacgcagatgtcctaaggggggctcatggagaacagaaatctccagtagaacaaaagggtaaaagcccccttga
@HWI-EAS293:3:1:30:671#0/1        -----------------------------------------AAGGGGGGCTCATGGAGAACAG--------------------------------------
@HWI-EAS293:3:1:25:1554#0/1       -----------------------------------------AAGGGGGGCTCATGGAGAACAG--------------------------------------
@HWI-EAS293:3:1:23:1620#0/1       -----------------------------------------AAGGGGGGCTCATGGAGAACAG--------------------------------------
@HWI-EAS293:3:1:14:103#0/1        -----------------------------------------AAGGGGGGCTCATGGAGAACAG--------------------------------------
@HWI-EAS293:3:1:20:1936#0/1       -----------------------------------------AAGGGGGGCTtATGGAGAACA---------------------------------------
@HWI-EAS293:3:1:20:1062#0/1       -----------------------------------------AAGGGGGGCTCATGGAGAACA---------------------------------------
@HWI-EAS293:3:1:18:589#0/1        -----------------------------------------AAGGGGGGCTCATGGAGAACA---------------------------------------
@HWI-EAS293:3:1:8:1585#0/1        -----------------------------------------AAGGGGGGCTCATGGAGAAC----------------------------------------
@HWI-EAS293:3:1:8:1353#0/1        -----------------------------------------AAGGGGGGCTCATGGAGAAC----------------------------------------
@HWI-EAS293:3:1:8:102#0/1         -----------------------------------------AAGGGGGGCTCATGGAGAAC----------------------------------------
@HWI-EAS293:3:1:50:1843#0/1       -----------------------------------------AAGGGGGGCTCATGGAGAAC----------------------------------------
@HWI-EAS293:3:1:46:1079#0/1       ---------------------------AACGCAGATGTCCTAAGGGGGGCTCATGGAGAAC----------------------------------------
@HWI-EAS293:3:1:44:1715#0/1       -----------------------------------------AAGGGGGGCTCATGGAGAAC----------------------------------------
@HWI-EAS293:3:1:43:286#0/1        -----------------------------------------AAGGGGGGCTCATGGAGAAC----------------------------------------
@HWI-EAS293:3:1:43:1481#0/1       -----------------------------------------AAGGGGGGCTCATGGAGAAC----------------------------------------
@HWI-EAS293:3:1:42:553#0/1        -----------------------------------------AAGGGGGGCTCATGGAGAAC----------------------------------------
@HWI-EAS293:3:1:38:1799#0/1       -----------------------------------------AAGGGGGGCTCATGGAGAAC----------------------------------------
@HWI-EAS293:3:1:36:2041#0/1       -----------------------------------------AAGGGGGGCTCATGGAGAAC----------------------------------------
@HWI-EAS293:3:1:36:1122#0/1       -------------------------------------------GGGGGGCTCATGGAGAAC----------------------------------------
@HWI-EAS293:3:1:35:1685#0/1       -----------------------------------------AAGGGGGGCTCATGGAGAAC----------------------------------------
@HWI-EAS293:3:1:34:1802#0/1       -----------------------------------------AAGGGGGGCTCATGGAGAAC----------------------------------------
@HWI-EAS293:3:1:34:1194#0/1       -----------------------------------------AAGGGGGGCTCATGGAGAAC----------------------------------------
@HWI-EAS293:3:1:33:1042#0/1       -----------------------------------------AAGGGGGGCTCATGGAGAAC----------------------------------------
@HWI-EAS293:3:1:31:809#0/1        -----------------------------------------AAGGGGGGCTCATGGAGAAC----------------------------------------
@HWI-EAS293:3:1:31:769#0/1        -----------------------------------------AAGGGGGGCTCATGGAGAAC----------------------------------------
@HWI-EAS293:3:1:31:1226#0/1       -----------------------------------------AAGGGGGGCTtATGGAGAAC----------------------------------------
@HWI-EAS293:3:1:30:1768#0/1       -----------------------------------------AAGGGGGGCTCATGGAGAAC----------------------------------------
@HWI-EAS293:3:1:26:774#0/1        -----------------------------------------AAGGGGGGCTCATGGAGAAC----------------------------------------
@HWI-EAS293:3:1:23:1799#0/1       -----------------------------------------AAGGGGGGCTCATGGAGAAC----------------------------------------
@HWI-EAS293:3:1:22:842#0/1        -----------------------------------------AAGGGGGGCTCATGGAGAAC----------------------------------------
@HWI-EAS293:3:1:22:1042#0/1       -----------------------------------------AAGGGGGGCTCATGGAGAAC----------------------------------------
@HWI-EAS293:3:1:20:1481#0/1       -----------------------------------------AAGGGGGGCTCATGGAGAAC----------------------------------------
@HWI-EAS293:3:1:19:1189#0/1       -----------------------------------------AAGGGGGGCTCATGGAGAAC----------------------------------------
@HWI-EAS293:3:1:18:1985#0/1       -----------------------------------------AAGGGGGGCTCATGGAGAAC----------------------------------------
@HWI-EAS293:3:1:18:1593#0/1       -----------------------------------------AAGGGGGGCTCATGGAGAAC----------------------------------------
@HWI-EAS293:3:1:16:771#0/1        -----------------------------------------AAGGGGGGCTCATGGAGAAC----------------------------------------
@HWI-EAS293:3:1:16:209#0/1        -----------------------------------------AAGGGGGGCTCATGGAGAAC----------------------------------------
@HWI-EAS293:3:1:15:825#0/1        -----------------------------------------AAGGGGGGCTCATGGAGAAC----------------------------------------
@HWI-EAS293:3:1:14:49#0/1         -----------------------------------------AAGGGGGGCTCATGGAGAAC----------------------------------------
@HWI-EAS293:3:1:13:1348#0/1       -----------------------------------------AAGGGGGGCTtATGGAGAAC----------------------------------------
@HWI-EAS293:3:1:10:620#0/1        -----------------------------------------AAGGGGGGCTCATGGAGAAC----------------------------------------
@HWI-EAS293:3:1:10:1265#0/1       -----------------------------------------AAGGGGGGCTCATGGAGAAC----------------------------------------
@HWI-EAS293:3:1:46:834#0/1        --------------------------------AGATGTCCTAAGGGGGGCTC-------------------------------------------------
@HWI-EAS293:3:1:23:1674#0/1       --------------------------------AGATGTCCTAAGGGGGGCTC-------------------------------------------------
@HWI-EAS293:3:1:21:1444#0/1       --------------------------------AGATGTCCTAAGGGGGGCTC-------------------------------------------------
@HWI-EAS293:3:1:15:1821#0/1       ------------------------------------GTCCcAAGGGGGGCTC-------------------------------------------------
```
